# The mTORC2 signaling network: targets and cross-talks

**DOI:** 10.1042/BCJ20220325

**Published:** 2024-01-25

**Authors:** Aparna Ragupathi, Christian Kim, Estela Jacinto

**Affiliations:** Department of Biochemistry and Molecular Biology, Robert Wood Johnson Medical School, Rutgers University, New Brunswick, NJ, U.S.A.

**Keywords:** Akt, cell growth, mTOR, mTORC2, metabolism, rictor, SIN1

## Abstract

The mechanistic target of rapamycin, mTOR, controls cell metabolism in response to growth signals and stress stimuli. The cellular functions of mTOR are mediated by two distinct protein complexes, mTOR complex 1 (mTORC1) and mTORC2. Rapamycin and its analogs are currently used in the clinic to treat a variety of diseases and have been instrumental in delineating the functions of its direct target, mTORC1. Despite the lack of a specific mTORC2 inhibitor, genetic studies that disrupt mTORC2 expression unravel the functions of this more elusive mTOR complex. Like mTORC1 which responds to growth signals, mTORC2 is also activated by anabolic signals but is additionally triggered by stress. mTORC2 mediates signals from growth factor receptors and G-protein coupled receptors. How stress conditions such as nutrient limitation modulate mTORC2 activation to allow metabolic reprogramming and ensure cell survival remains poorly understood. A variety of downstream effectors of mTORC2 have been identified but the most well-characterized mTORC2 substrates include Akt, PKC, and SGK, which are members of the AGC protein kinase family. Here, we review how mTORC2 is regulated by cellular stimuli including how compartmentalization and modulation of complex components affect mTORC2 signaling. We elaborate on how phosphorylation of its substrates, particularly the AGC kinases, mediates its diverse functions in growth, proliferation, survival, and differentiation. We discuss other signaling and metabolic components that cross-talk with mTORC2 and the cellular output of these signals. Lastly, we consider how to more effectively target the mTORC2 pathway to treat diseases that have deregulated mTOR signaling.

## Introduction

The cellular target of the natural compound, rapamycin, was first discovered in the budding yeast and named TOR (target of rapamycin) [1–3]. The identification of the mammalian counterpart, mTOR (aka FRAP, RAFT, RAPT), which is also inhibited by rapamycin, soon followed [4–6]. Rapamycin, an antibiotic that is produced by the bacterium *Streptomyces hygroscopicus*, forms a complex with the prolyl isomerase FKBP12 and targets the FKBP-rapamycin binding region in TOR/mTOR to allosterically inhibit this protein kinase. Rapamycin and its analogs (rapalogs) are now used in the clinic to treat a variety of diseases including tuberous sclerosis, lymphangioleiomyomatosis (LAM), and certain cancers [7–9]. It is also used as an immunosuppressant to prevent allograft rejection, in coronary stents to prevent restenosis, and is promising for the treatment of neurological disorders and as an anti-aging compound. Rapamycin inhibits or dampens the cellular functions of mTOR. However, it does not completely block TOR/mTOR functions. In yeast, TOR is encoded by two genes, TOR1 and TOR2. Whereas loss of TOR1 alone has little effect on growth, loss of TOR2 is lethal and cannot be overcome even with TOR1 overexpression. TOR1 and TOR2 have a rapamycin-sensitive redundant function in G1 cell cycle progression, whereas TOR2 has a rapamycin-insensitive unique function in actin cytoskeleton reorganization [10–12]. These findings hinted that TOR could be modulated by partnering with different proteins and thereby launched the quest to identify such proteins. Two distinct TOR protein complexes, mTOR complex 1 (mTORC1) and mTORC2, were identified simultaneously in yeast and mammals [13–20]. In higher eukaryotes, mTOR is encoded only by one gene yet these protein complexes are also conserved. Yeast TORC1 and mammalian mTORC1 bind and are inhibited by the rapamycin/FKBP12 complex. In contrast, mTORC2 is insensitive to rapamycin. Structural studies reveal that this insensitivity is due to occlusion of the rapamycin/FKBP12 binding region in this protein complex, hence the inability of mTORC2 to directly associate with rapamycin [21–23]. However, prolonged exposure to rapamycin indirectly inhibits mTORC2 since this compound can target newly synthesized mTOR, thus preventing *de novo* mTORC2 assembly [24].

**Figure 1.**
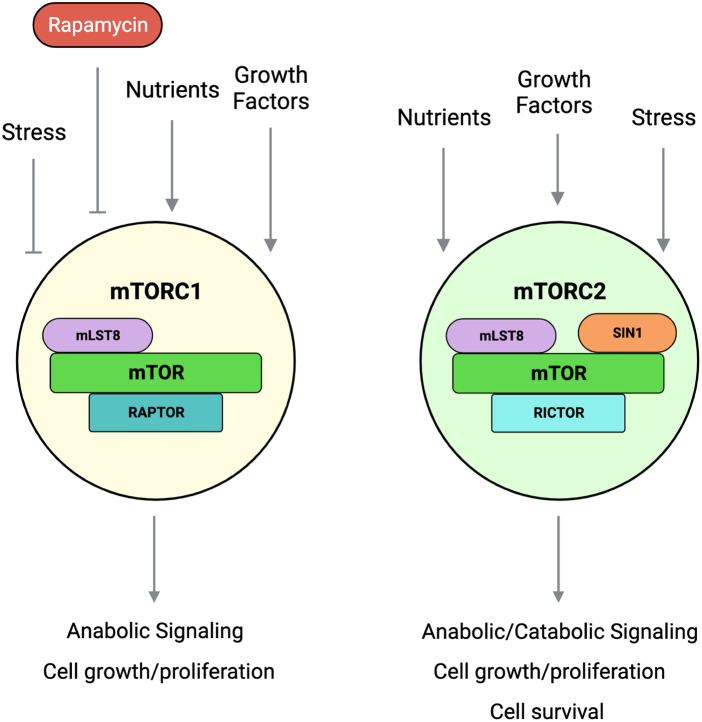
Two mTOR protein complexes respond to environmental signals to promote cell growth. mTORC1, which is sensitive to rapamycin, is activated by nutrients and growth factors to promote anabolic signaling and cell growth/proliferation. mTORC2, which is insensitive to rapamycin, is activated by stress to enhance cell survival and by nutrients and growth factors to promote cell growth and proliferation.

Studies on mTORC1 have been expedited in various species and the context of diseases due to the availability of rapamycin. There are ongoing efforts to develop specific and effective mTORC2 inhibitors. Studies to delineate mTORC2 functions have relied mainly on genetic manipulation of mTORC2 components and pharmacological inhibitors that block mTOR (mTORC1/mTORC2) activities. Early studies that knocked out each of the mTORC2 components, rictor, SIN1, and mLST8 in mice support that these proteins are essential during development and that they mediate mTORC2 function in substrate phosphorylation [18,25,26]. Compared with mTORC1, which has a predominantly anabolic role, the function of mTORC2 seems to be more diverse and this complex is activated by the presence of growth signals as well as stress conditions (Figure 1). The growth-related functions of mTORC2 are conserved from yeast to humans and its stress-related function is critical to allow metabolic reprogramming and favor cell survival. In this review, we discuss the regulation and targets of mTORC2 and how it intersects with other cellular pathways to modulate responses to growth and stress stimuli.

## mTORC2 composition and structure

**Figure 2.**
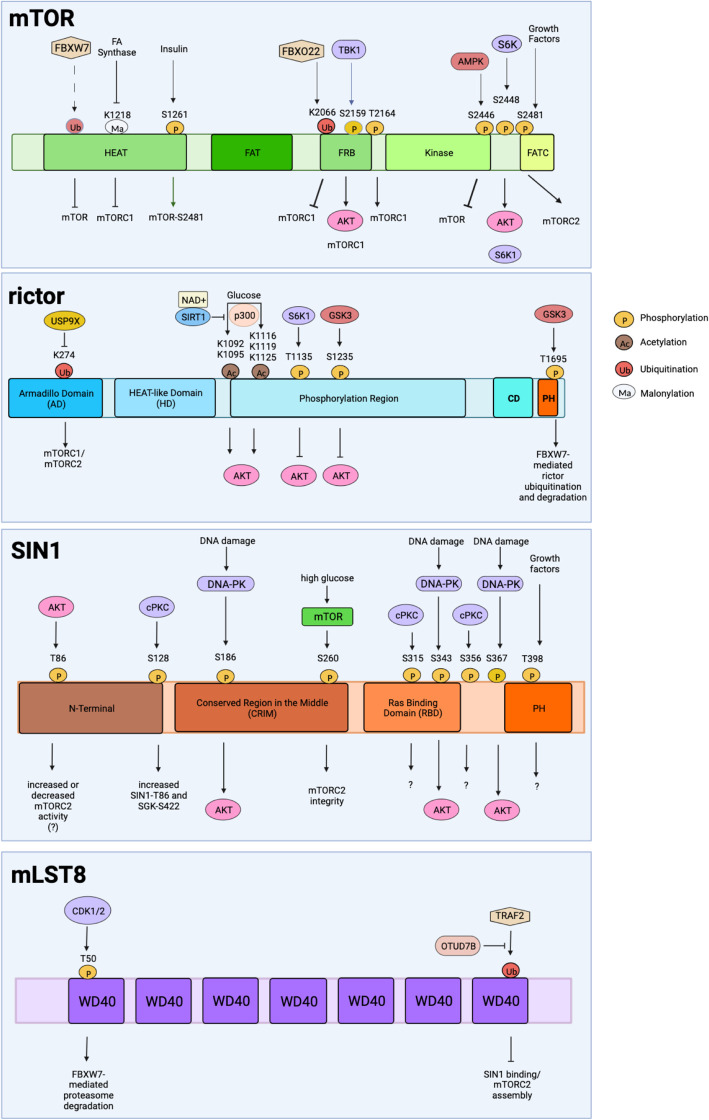
mTORC2 complex component structural domains and their post-translational modifications. mTORC2 components undergo post-translational modifications including phosphorylation, acetylation, ubiquitination, and malonylation at the indicated amino acid residues. The signals or upstream regulators of such modifications are indicated above the modification sites. The corresponding effect of each modification is indicated directly below.

mTORC2 is composed of the catalytic mTOR protein kinase subunit and the conserved components, rapamycin-insensitive companion of mTOR, rictor (AVO3 in *Saccharomyces cerevisiae*) [13,16,17], and the stress-activated protein kinase-interacting protein 1, SIN1 (aka MAPKAP1, MIP1; AVO1 in *S. cerevisiae*) [18,19,20] (Figure 2). It also associated with mLST8 (mammalian lethal with SEC13 [8]), which is conserved in yeast, and Protor (protein observed with mTOR), which is found only in higher eukaryotes. Structural and biochemical studies reveal that mTORC2, like mTORC1, exists as dimers of their multi-subunit complexes ([27,28]). The overall shape of mTORC2 resembles a three-dimensional rhombus [21–23]. Interactions among the mTORC2 components cause the units to be packed tightly compared with mTORC1 [23]. Since SIN1 has different isoforms [20,29], mTORC2 likely exists in different versions.

mTOR is an atypical protein kinase belonging to the family of PI3K (phosphatidylinositol 3-kinase)-related kinases (PIKK). It contains specific structural domains that modulate its function and facilitate its activity. At the amino terminus, there are over 30 consecutive HEAT repeats that structurally form α-solenoids [30]. The HEAT repeat, consisting of ∼40 amino acids, was first found in Huntingtin, Elongation factor 3, A subunit of PP2A, TOR. The PI3K-related bilobal (N- and C-lobe) kinase domain is preceded by the ∼600 residue FAT (FRAP, ATM, TRRAP) domain and the FRB (FKBP12/rapamycin binding) domain. The active site is highly recessed due to the FRB region and the catalytic site has restricted access [31]. A ∼40 residue insertion (termed LBE) in the C-lobe of the kinase domain serves as a binding site for mLST8 [31]. The FAT domain forms the I-site, an inositol hexakisphosphate (InsP6) pocket which is not specifically involved in modulating mTORC2 activity but is necessary for protein folding or complex assembly [30]. The mTOR interactor, DEPTOR, via its PDZ domain, also binds to the FAT domain of mTOR [32]. The FATC (FAT C-terminal) domain at its C terminus interacts with the activation loop and may thus stabilize its structure.

Before the assembly of mTOR complexes, the folding and maturation of mTOR (as well as other related PIKKs) is facilitated by the Tel2–Tti1–Tti1 (TTT) complex, in collaboration with the heat shock proteins, Hsp90 and Hsp70 [33–35]. Studies using yeast TOR (and other PIKK) reveal that TTT binds to and maintains nascent PIKKs in a non-native state during synthesis of the C-terminal domains FAT and KIN (which form the highly conserved FATC region that is crucial for proper folding [36]. These studies indicate that mTOR/PIKK biogenesis is coupled to translation but the assembly of mTOR complex occurs post-translationally.

Rictor consists of several groups of α helices that form multiple interactions with mTOR [30]. Rictor contains three domains: the N-terminal armadillo domain (AD), the HEAT-like domain (HD), and the C-terminal domain (CD). The AD consists of nine repeats of three α helices which segregate the HD and CD. The CD comprises four α-helix bundles [30]. The HD and CD flank a phosphorylation region, which harbors most of the phosphorylation sites identified for rictor [37]. The AD forms contact with mTOR, whereas the CD occludes the FRB [23]. Rictor and SIN1 interact with the FRB domain of mTOR in a manner that could prohibit the binding of rapamycin/FKBP12 as well as other mTORC1 substrates including 4EBP1 and S6K [23]. Similar to raptor, rictor binds to mTOR at the HEAT region. Computational analysis also predicts that rictor contains pleckstrin homology (PH) domains [38], which bind phosphoinositides and could facilitate membrane localization. There is also sequence homology to mammalian ribosomal 39S protein L17, suggesting that this region may allow interaction with ribosomes [38]. Whether these predicted regions modulate rictor functions needs to be investigated. Rictor's HD consists of an A-site that binds ATP [30]. The role of ATP binding to this site is still unclear but so far it does not seem to allosterically affect mTORC2 activity.

**Figure 3.**
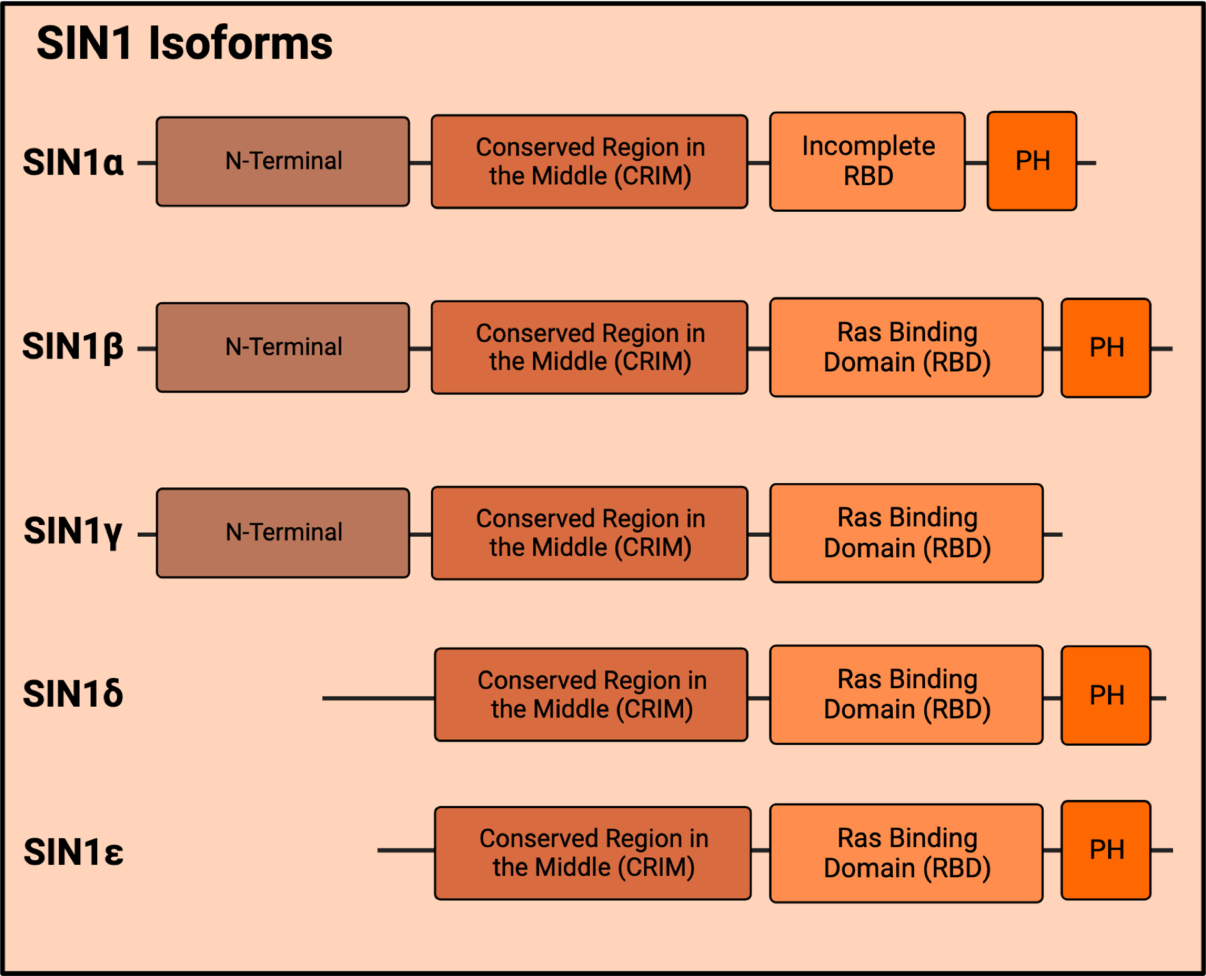
SIN1, a component of mTORC2, exists as multiple isoforms due to alternative splicing of transcripts. All isoforms contain the CRIM domain, which plays a role in mTORC2 substrate recruitment.

Another subunit of mTORC2, SIN1, is encoded by six splice variants thus resulting in SIN1 isoforms (Figure 3) [20,39]. Whereas SIN1α, β, and γ assemble individually with mTORC2, SIN1δ and SIN1ε lack the N-terminal domain that is present in the other SIN1 isoforms. SIN1δ, and likely SIN1ε as well, do not associate with mTORC2. When each of the isoforms is expressed in SIN1-deficient murine embryonic fibroblasts, only SIN1α and SIN1β strongly restore, whereas SIN1γ weakly restores mTORC2 signaling [20,39]. Distinct regulation and functions of SIN1 isoforms remain to be characterized. Structurally, SIN1 is found near the cleft of mTOR where catalysis occurs. It interacts with rictor and links rictor to mLST8, thus crucial for mTORC2 assembly. SIN1 contains four regions: the N-terminal region, the conserved region in the middle (CRIM; conserved among SIN1 family and present in all of its splice variants) [40], the Ras-binding domain (RBD), and the PH domain [23]. The N-terminal region of SIN1 is predominantly involved in the interactions between SIN1 and rictor. This region of SIN1 is located where the ARM domain and HEAT-like domain of rictor meet. The conformation of this N-terminal part is altered in the presence of SGK1, but not Akt [41]. This region also includes Gln68, which is required for the activation of SGK1, but not Akt or PKC [42]. Nonetheless, the other three domains are more readily available for other interactions compared with the N-terminus [30]. Additionally, SIN1 contacts the FRB of mTOR. SIN1 recruits mTORC2 substrates, such as Akt, PKC, and SGK, whereby its CRIM domain (aa 137–266) is necessary for mTORC2 to bind to its substrates [22,30,43]. The active site of mTORC2 is obstructed by the CRIM area of AVO1/SIN1 found on the border near the crevice [22]. This domain contains a fold that is similar to that of ubiquitin with a chain of amino acids containing acidic residues that provide interactions with key mTORC2 substrates [21,44]. The positioning of the CRIM domain is also affected by mLST8 ubiquitination at Lys305 and Lys313 [30,45]. The SIN1-RBD interacts with Ras and this interaction affects Ras/ERK pathway activation depending on the stimulus [46]. The PH domain of SIN1 has an inhibitory effect on SIN1–Ras binding [46,47]. The SIN1 isoform (SIN1γ) that is devoid of the PH domain associates more strongly with Ras. Both AVO1 (*S. cerevisiae* SIN1) and SIN1 have been shown to bind phosphoinositides. In particular, phosphatidylinositol (3,4,5) trisphosphate [PtdIns(3,4,5)P3 or PIP3] binding to the SIN1-PH relieves its inhibition of the mTOR kinase domain by allowing substrate access, thus promoting mTORC2 activity [48–50]. Loss of this inhibitory effect of SIN1-PH on mTOR increases mTORC2 signaling and occurs in cancer patient-derived mutations of SIN1-PH (D412G and R81T), rendering this SIN1 mutant oncogenic [49,51].

mLST8 is made of seven WD40 repeats in the form of a β-propeller. Structural studies indicate that mLST8 could interact with mTOR across six of the seven WD40 repeats [31]. Mutation of four (Q44E, N46L, W272F, and W274F) mLST8 residues that are predicted to interact with mTOR disrupts mTORC2 integrity and activity [52]. mLST8 also helps SIN1 position its CRIM domain for substrate recruitment [30]. Although mLST8 is found in both mTORC1 and mTORC2, it is essential for mTORC2 assembly and activity [26]. The chaperonin complex, CCT (chaperonin-containing TCP1), interacts with mLST8 and assists in its folding [53]. Enhancing the translation of CCT4, a component of the CCT chaperonin complex, increases the expression of mLST8 by promoting its folding [54].

In higher eukaryotes, mTORC2 associates with other less evolutionarily conserved proteins. Protor, which has two isoforms Protor1 (PRR5) and Protor2 (PRR5L), is found in mTORC2 although it is not required for the assembly of the core complex [55–57]. Loss of both Protor1 and Protor2 does not affect mTORC2 integrity and phosphorylation of Akt and PKCα. Instead, loss of Protor1/2 reduces the phosphorylation of SGK1 and the SGK1 substrate NDRG1 (N-Myc down-regulated gene 1), specifically in the kidney. Another mTOR-interacting protein, DEPTOR, which contains tandem N-terminal DEP (Dishevelled, Egl-10, Pleckstrin) domain and a C-terminal PDZ (PSD95, DlgA, Zo-1) domain is found in both mTORC2 as well as mTORC1 [32]. The PDZ domain and the DEP domain tandem (DEPt) interact with mTOR [58]. Whereas the PDZ domain creates a tight association and mildly activates mTOR, it also anchors DEPt association to mTOR. The latter allosterically inhibits mTOR. Hence, the structure of DEPTOR could provide clues as to why there are discrepant findings on its role in mTORC2 signaling [59]. Several studies support that DEPTOR negatively regulates mTORC1 [32,60,61]. High DEPTOR expression in multiple myeloma down-regulates mTORC1 signaling, thus relieving the mTORC1-mediated negative feedback to growth factor/PI3K signaling. This results in the maintenance of PI3K/Akt signaling, suggesting that DEPTOR could indirectly activate mTORC2 [32]. In another context, its disruption promotes both mTORC1 and mTORC2 signaling, suggesting it serves as a negative regulator of both complexes [62,63]. Further studies to determine how the DEPTOR domains are modulated would shed light on how DEPTOR could precisely regulate mTORC2. Another mTORC2 interactor is Sestrin2, which binds via GATOR2. Depletion of the GATOR2 component, WDR24 or WDR59, abolishes Sestrin2-induced Akt activation [64]. Whereas Sestrin2 suppresses mTORC1, it positively modulates mTORC2 [64,65].

Other signaling molecules also interact with mTORC2 complex components. The cyclin-dependent kinase 9 (CDK9) binds to rictor, SIN1, and mLST8 in the cytoplasm and modulates mRNA translation via phosphorylation of the translation regulators LARP1 and rpS6 [66]. The precise function of mTORC2 in this complex remains to be examined but targeting this complex leads to antileukemic responses in acute myeloid leukemia xenografts. The mTORC2 components rictor and SIN1 also bind to other proteins independently of mTOR. Rictor is associated with the integrin-linked kinase (ILK) [67]. Depletion of rictor, but not mTOR, diminishes the levels of Akt-Ser473 phosphorylation that are associated with the ILK complex, suggesting that rictor/ILK could mediate Akt phosphorylation in specific contexts. In cultured adipocytes, rictor forms a complex with Myo1c, a molecular motor protein that is involved in cortical actin remodeling [68]. Disruption of this complex does not affect Akt-Ser473 phosphorylation but diminishes the phosphorylation of the actin filament regulatory protein, paxillin, at Tyr118 and abrogates Myo1c-induced membrane ruffling. It is not clear which kinase could mediate this rictor/Myo1c-dependent phosphorylation.

## Transcriptional regulation of mTORC2

The expression of mTORC2 components can be modulated at the level of transcription. Several transcription factors increase mTOR transcription including sirtuin 1 (SIRT1) [69], myeloid zinc finger 1 (MZF1) [70], and Nrf2 [71] while Ikaros represses mTOR transcription in B cell acute lymphoblastic leukemia [72]. The rictor promoter has an NF-κB response element (−301 to −51 bp) [73]. The NF-κB component, p65, binds to this response element to enhance rictor expression upon stimulation with the proinflammatory cytokine, TNFα. This NF-κB-mediated rictor up-regulation contributes to the metastasis of renal cell carcinoma. mLST8 transcription is modulated by STAT3 and its promoter has potential STAT3-binding sites at regions −951 to −894 [74]. This regulation by STAT3 plays a role in cap-dependent translation in cancer cells. So far, little is known as to how these transcription factors could regulate mTORC2 component expression in response to cellular stimuli.

The stability and translation of mRNAs of mTORC2 components are also regulated. A splice variant of mTOR with a molecular mass of 80 kDa, which is less than the full-length mTOR (289 kDa), has been reported to promote G1 cell cycle progression and cell proliferation [75]. It is unclear if other mTOR splice variants exist and regulation of expression and functions of such variants would need to be examined. The RNA-binding protein nucleolin binds to *mTOR* mRNA and transports it to injured neuronal axons, which then up-regulates mTOR by increasing local mTOR translation [76]. Deletion of the 3′UTR of *mTOR* mRNA leads to its impaired axonal localization and translation, thus preventing neuron survival after injury. This localized mTOR translation also promotes compartmental translation of retrograde injury signaling molecules such as importin β1 and STAT3. An RNA processing and ribosome biogenesis protein, SMN (survival motor neuron), also associates with mTOR transcript and its deficiency is linked to increased nuclear retention of mTOR mRNA [77]. Another RNA-binding protein AUF1 also associates with mRNAs of mTORC2 components including mTOR, rictor, and SIN1 as well as the mTORC2 targets Akt and GFAT1 [78]. It remains unclear how AUF1 could modulate mTORC2 levels or signaling. Given its role in promoting Akt membrane localization to allow its phosphorylation in this compartment, AUF1 may also promote colocalization of mTORC2 with Akt.

There is accumulating evidence on the regulation of rictor expression at the level of mRNA expression and stability. Rictor pre-mRNA splicing and maturation are regulated by the ubiquitin-specific protease 39 (USP39), which is involved in assembling the mature spliceosome complex [79]. USP39 expression is up-regulated in cancers such as human esophageal squamous cell carcinoma (ESCC). By mediating increased rictor expression, which enhances mTORC2 activity, USP39 could contribute to tumor progression. The long 3′UTR of rictor has built-in cis-regulatory elements that affect both mRNA stability and translational control. This region has four consensus binding sites for RNA-binding protein HuR, which affects mRNA integrity [80]. These sites may in turn respond to mTORC2 signaling, as mTORC2 activates heat shock transcription factor HSF1, which induces HuR activity in glioblastoma (GBM) models. Thus, a feed-forward loop is generated by mTORC2/Akt and HSF1/HuR, resulting in elevated mTORC2 activity in GBM tumors. Several studies suggest that the expression of rictor could be modulated by micro-RNAs (miR) in different tissues. Most of these miRs decrease rictor mRNA levels. miR-497, miR-218, miR-153, miR-429, and miR-196b inhibit rictor expression to prevent cell growth or angiogenesis in hepatocellular carcinoma, prostate cancer, glioma, NK cell lymphoma, and melanoma models [70,81–85]. Controlling rictor mRNA levels could also be important during cellular differentiation and metabolic reprogramming. miR-188 targets rictor to control the transition between osteogenesis and adipogenesis in bone marrow mesenchymal stem cells [86]. miR-582 diminishes rictor expression, thus down-regulating mTORC2 signaling and regulates metabolism in early B cell development [87]. miRNA-342-3p directly targets rictor in T-regulatory (Treg) cells and reprograms metabolism in response to glucocorticoids [88]. Targeting the specific expression of various micro-RNAs in different tissues could be promising for selective blockade of mTORC2 activity.

SIN1 has several isoforms and its transcripts use alternative polyadenylation signals, resulting in several splice variants (Figure 3) [20,29,40]. The SIN1 5′UTR is long (at least 330 nucleotides) and highly structured, suggesting elaborate post-transcriptional regulation [40]. In colon carcinoma, SIN1 translation is controlled by programmed cell death 4 protein (Pdcd4), which inhibits the translation initiation factor 4A (eIF4A). The 5′ UTR of SIN1 is necessary for this function of Pdcd4. In colon cancer cells, loss of Pdcd4 increases SIN1 protein levels and promotes tumor invasiveness [89]. SIN1 expression is also modulated by non-coding RNAs. The lncRNA, CASC9-1 (cancer susceptibility candidate 9) enhances SIN1 expression and promotes malignancy in cervical squamous cell carcinoma (CSCC) by directly targeting miR-383-5p, a negative regulator of SIN1 [90]. A key question to address would be how the transcripts of different SIN1 isoforms could be regulated in response to different stimuli.

Protor1/PRR5 expression is increased via the long non-coding RNA LINC01133, which antagonizes the role of the RNA-binding protein hnRNPA2B1, a negative mRNA regulator of PRR5 [91]. Protor1 up-regulation in triple-negative breast cancer (TNBC) increases Akt activation and has pro-tumorigenic role in TNBC. Protor2 also associates with an RNA-binding protein, tristetraprolin (TTP) and its binding stimulates TTP-mediated mRNA turnover of several mRNAs that are regulated by TTP [92]. Whether Protor2 expression is also modulated by RNA-binding proteins remains to be examined.

Increased mTORC2 activation is often characterized by up-regulation of rictor and/or SIN1 expression. Elucidating the specific mechanisms involved during their elevated expression should unravel more selective strategies to inhibit mTORC2 in cancer and other diseases.

## Post-translational modification of mTORC2 components

Components of mTORC2 undergo post-translational modifications that can affect their expression, localization, mTORC2 integrity, and kinase activity (Figure 2). mTOR itself is phosphorylated at Ser2446/2448 [93–95]. Phosphorylation at Ser2448 correlates with increased phosphorylation of mTORC1 and mTORC2 substrates S6K1 and Akt, respectively. This site is reciprocally phosphorylated by S6K1, consistent with the sensitivity of this phosphosite to rapamycin treatment and amino acid deprivation [93,96]. Phosphorylation at Ser2446 correlates with nutrient deprivation and increased AMPK activity [97]. When Ser2448 is phosphorylated, phosphorylation at Ser2446 via AMPK is diminished. How Ser2446 and Ser2448 phosphorylation could be coordinately regulated in response to growth signals deserves further investigation. Growth factor signaling enhances the phosphorylation of mTOR at Ser2481, a site phosphorylated by mTOR itself, both as part of mTORC1 or mTORC2 [98–100]. Autophosphorylation at Ser2481 is linked to intact mTORC2 signaling [99–101]. Other mTOR phosphorylation sites include Ser2159 and Thr2164, which regulate mTOR and Raptor binding, while Ser1261 phosphorylation increases mTOR activity in response to insulin and is critical for Ser2481 phosphorylation [102,103]. Ser2159 is phosphorylated by TBK1 (TANK-binding kinase 1), a host innate immune kinase [104,105]. This phosphorylation activates both mTORC1 and mTORC2. It promotes the mTORC2-mediated phosphorylation of Akt in response to growth factor and poly (I:C), a viral dsRNA mimetic. Furthermore, diet-induced obese mice that express the mutant mTOR-Ser2159Ala share phenotypic similarities as mice with specific inactivation of mTOR complexes, including exacerbated hyperglycemia and systemic insulin resistance [106]. These findings support the role of this phosphorylation in mTOR signaling. The precise contribution of this phosphorylation to mTORC2 signaling would need to be addressed. Whether there are phosphorylation sites in mTOR that could exclusively modulate mTORC2 activity also remains to be investigated.

mTOR also undergoes ubiquitination and malonylation. The tumor suppressor and E3 ubiquitin ligase, FBXW7, ubiquitinates mTOR at a consensus CDC (cell division cycle) phosphodegron sequence in its HEAT domain, promoting its degradation. Whether mTOR ubiquitination affects mTORC2 is unclear but so far *Fbxw7*^−/−^ MEFs have increased S6K phosphorylation but no change in Akt phosphorylation [107]. mTOR ubiquitination at Lys2066, mediated by another F-box protein, FBXO22, does not promote mTOR degradation but hinders substrate recruitment of mTORC1 [108]. This ubiquitination occurs in response to the accumulation of uncharged tRNAs during amino acid starvation and is mediated by the phosphorylation of FBXO22 by activated GCN2. While Akt phosphorylation by mTORC2 was not diminished by the FBXO22-mediated mTOR ubiquitination, its role on mTORC2 signaling during starvation remains to be interrogated. Malonylation, a lysine modification that uses malonyl-CoA as a substrate, also affects mTOR activity. In response to fatty acid synthase inhibition in endothelial cells, mTOR undergoes malonylation at K1218. This ultimately decreases mTORC1 activity, suggesting that this modification impairs mTOR anabolic function. It is unclear whether malonylation directly affects mTORC2 activity as fatty acid synthase inhibition does not appreciably change Akt phosphorylation [109].

Post-translational modifications in rictor and SIN1 are abundant and serve to regulate mTORC2 activity. Based on MS/MS analysis or motif analysis predictions, rictor has ∼37 putative phosphorylation sites located mainly at the C-terminal region [37,110]. So far, the characterized phosphosites in rictor negatively regulate mTORC2 activity. This would support the notion that mTOR as part of mTORC2 has constitutive activity and that modification of mTOR partners modulates its activity. One of these rictor phosphosites, Thr1135, is phosphorylated by S6K to negatively regulate mTORC2. Phosphodeficiency at Thr1135 leads to increased Akt phosphorylation with no observable change in SGK or cPKC phosphorylation nor mTORC2 activity [37,110,111]. Furthermore, increased Thr1135 phosphorylation enhances the binding of rictor to 14-3-3 proteins, which consequently down-regulates mTORC2 activity. Decreased rictor Thr1135 phosphorylation also occurs during PTEN depletion, leading to increased mTORC2 complex formation and signaling [112]. Together, these findings support that Thr1135 phosphorylation negatively regulates mTORC2 activity. Rictor also undergoes GSK3-dependent phosphorylation at Thr1695, which is located in a putative CDC4 phosphodegron (CPD) motif within rictor. Notably, Thr1695 site mutations block rictor interaction FBXW7, which regulates ubiquitination and degradation of both mTOR and rictor [113]. Hence, Thr1695 phosphorylation down-regulates mTORC2 activity. During cell stress, GSK3 also phosphorylates rictor at Ser1235, which blocks Akt-mTORC2 binding and impairs mTORC2 activity [114]. Since GSK3 is negatively regulated by Akt, these findings suggest that as PI3K/Akt signaling wanes, GSK3 is de-repressed and thus promotes Thr1695 and/or Ser1235 phosphorylation, consequently diminishing mTORC2 activity. The functions of the other putative rictor phosphorylation sites warrant further investigation.

Acetylation at various sites in rictor also impacts mTORC2 activity [115]. Deletion mutants of rictor that abolish Lys residues K1092/K1095 and K1116/K1119/K1125 reduce mTORC2 activity [116]. This multisite acetylation region is adjacent to a stabilization region, which is necessary for interaction with SIN1 and mLST8 [115]. Rictor acetylation is mediated by p300 while deacetylase inhibitors, such as the HDAC inhibitors, sustain rictor acetylation and mTORC2 activity. SIRT1, which is responsive to the nutrient NAD+ (nicotinamide adenine dinucleotide), also deacetylates rictor, suggesting that mTORC2 is modulated by the availability of this nutrient [115]. Glucose or acetate increases rictor acetylation, which amplifies mTORC2 activity in GBM and confers resistance to EGFR-, PI3K-, or Akt-targeted therapies [116]. In HepG2 cells, enhanced rictor deacetylation through SIRT1 inhibits mTORC2 activity [117]. Restoring rictor acetylation in GCN5L1-deprived cardiac cells increases mTORC2 activation [118]. Hence, unlike Rictor phosphorylation, Rictor acetylation appears to predominantly increase mTORC2 activation while deacetylation inhibits mTORC2. Since acetylation relies on the availability of acetyl-CoA, future studies should determine how different nutrients that enhance the levels of this metabolite, such as glucose and lipids, affect rictor acetylation and mTORC2 function in metabolism.

Rictor undergoes ubiquitination via FBXW7 to promote its proteasome-mediated degradation [113]. Although the FBXW7-mediated ubiquitination site in rictor remains to be characterized, increased PI3K/Akt signaling suppresses rictor ubiquitination via preventing the GSK3/FBXW7 axis. These findings imply a feedback loop such that as mTORC2 activity is elevated, rictor ubiquitination is suppressed. The FBXW7-mediated degradation of rictor is also promoted by pharmacological Vitamin C (VitC) treatment of cancer cells [119]. VitC induces intracellular reactive oxygen species (ROS), which enhance the GSK3/FBXW7-mediated rictor ubiquitination, leading to the death of cancer cells. Rictor ubiquitination also occurs upon disruption of rictor/mTORC2 interaction with the transmembrane cell adhesion protein CD146 in endothelial cells. It is not clear what mediates rictor ubiquitination under these conditions but this modification down-regulates mTORC2 activity [120]. In contrast, rictor ubiquitination at Lys274 is associated with increased mTORC2 activity. This ubiquitin Lys63-linked modification is deubiquitinated by the ubiquitin-specific protease 9X (USP9X) [121,122]. Knockdown of USP9X enhances mTORC2 activity and accelerates the differentiation of myoblasts into myotubes [122]. Together, these findings demonstrate how rictor ubiquitination via different mechanisms controls mTORC2 activity.

Several phosphorylation sites have been identified in SIN1 but the function of most of these phosphosites remains to be further investigated. SIN1 is phosphorylated at Thr86 and Thr398. Phosphorylation of SIN1 at Thr86 occurs in the presence of growth factors [123]. This phosphorylation is mediated by Akt [123,124] although another study showed that S6K can also phosphorylate this site [51]. Whether SIN1-Thr86 phosphorylation enhances mTORC2 activity is controversial. SIN1-Thr86 phosphorylation leads to SIN1 dissociation from mTORC2, consequently inhibiting mTORC2 activity [51]. A SIN1-R8IT mutant allele that is deficient in phosphorylation at Thr86 found in ovarian cancer patients is linked to mTORC2 hyperactivation, suggesting that Thr86 phosphorylation has a negative regulatory function [51]. Furthermore, cryo-EM studies of mTORC2 revealed that SIN1-Thr86 is embedded in a negatively charged pocket of rictor, thus suggesting that phosphorylation of this site could be repulsive and speculatively promote mTORC2 disassembly [30]. However, another study demonstrated that SIN1-Thr86 phosphorylation is enhanced by Akt and serves as a positive feedback loop to augment mTORC2 activation [124]. Inhibition of the Ras-MAPK pathway in neuroblastoma also increases Akt activation via enhancing SIN1-Thr86 phosphorylation, supporting a positive relationship between SIN1 phosphorylation and mTORC2 activation [125]. Future studies should address how mTORC2 activity is affected by SIN1-Thr86 phosphorylation under different cellular conditions or contexts. SIN1 is also phosphorylated at Ser260 in an mTOR-dependent manner and this phosphorylation relies on high glucose levels and maintains the integrity of mTORC2 [126]. Three other sites in SIN1, Ser128, Ser315, and Ser356, undergo phosphorylation in a cPKC-dependent manner [127]. Ser128 is within a PKC recognition motif (KXS/TXK), whereas Ser356 is within a PKD motif (LXXRS/T). Ser315 resembles both PKC and PKD recognition motifs and is located within the RBD. Phosphorylation of these sites is not necessary for mTORC2 modulation of SGK1 or Akt, but Ser128 phosphorylation could allow SIN1 phosphorylation at Thr86, which could impact mTORC2 activity. In response to DNA damage, SIN1 is phosphorylated at SQ motifs including at Ser186, Ser343, and Ser367 [128]. DNA-PK phosphorylates the SIN1-SQ motifs and consequently enhances Akt-Ser473 phosphorylation during DNA damage. SIN1 phosphorylation at these motifs mediates its interaction with the BRCT (BRCA1 C-terminal) domains of ECT2, a guanine nucleotide exchange factor. Together, these findings reveal how SIN1 could be modulated by different protein kinases that cross-talk with mTORC2/Akt signaling.

mLST8 is shared between mTORC1 and mTORC2 but has direct effects on mTORC2 structure and activity. Loss of mLST8 prevents mTORC2 assembly while having little to no effect on mTORC1 [52]. mLST8 also interacts with SIN1 [21,52]. However, the mLST8–SIN1 interaction is interrupted when mLST8 undergoes K63 polyubiquitination through TRAF2 E3 ubiquitin ligase [45]. The ubiquitination of mLST8 by TRAF2 occurs on its WD7 where SIN1 also binds. In response, OTUD7B deubiquitinase removes mLST8's polyubiquitin chains to encourage SIN1 interaction and mTORC2 assembly. Polyubiquitination of mLST8 prevents SIN1 and rictor from binding to mTOR and instead favors raptor binding to form mTORC1. In contrast, deubiquitinated mLST8 interacts with SIN1 to form mTORC2 [45]. The deubiquitinase activity of OTUD7B is responsive to insulin and mLST8 ubiquitination is enhanced by IRS1/2 depletion, suggesting that the latter modulates OTUD7B activity [129]. Together, these findings reveal how mLST8 K63-linked polyubiquitination could be dynamically regulated by growth signals to control mTORC2 assembly. mLST8 is also ubiquitinated via FBXW7 to route it for proteasome-dependent degradation [130]. It is phosphorylated at Thr50 by CDK1 and phosphorylation at this site is nestled in the CDK1/2 consensus motif that is targeted by FBXW7. It is noteworthy that mLST8 is up-regulated in clear cell renal cell carcinoma and the FBXW7-mediated ubiquitination and degradation of mLST8 prevents tumor progression [130].

Collectively, the above findings illustrate how amino acid modifications in mTORC2 components regulate mTORC2 activity. As further studies unravel other modifications that utilize various nutrient metabolites, we will expand our understanding of how mTORC2 is modulated by nutrient fluctuations.

## mTORC2 cellular localization

**Figure 4.**
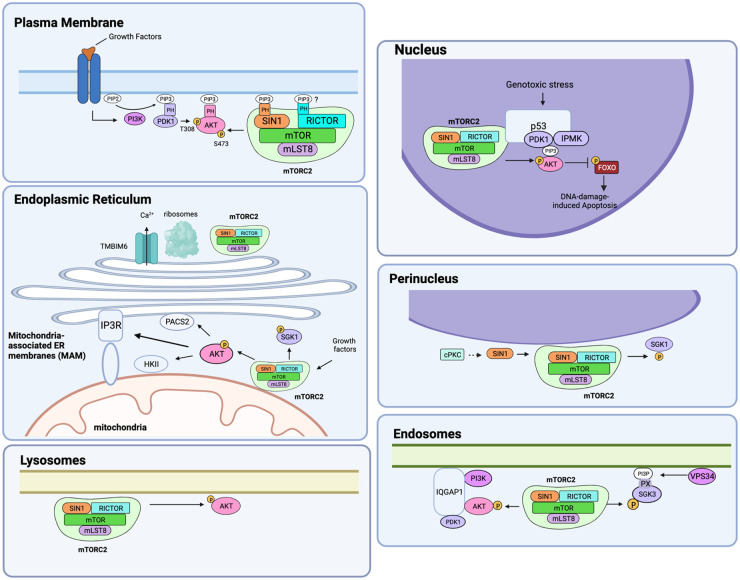
The colocalization of mTORC2 and its substrates in membrane and non-membrane compartments facilitates substrate phosphorylation. mTORC2 localizes to the plasma membrane, endoplasmic reticulum (ER), mitochondria-associated ER membranes (MAM), lysosomes, endosomes, and perinucleus as well as in the nucleus. In the periphery of the plasma membrane and possibly other membrane-containing compartments, its localization could be mediated by increased levels of phosphoinositides (such as PIP3 that binds to SIN1 or putatively rictor). There are also scaffolding proteins that could facilitate the recruitment of mTORC2 to cellular compartments, such as p53 in the nucleus.

Several studies support that mTORC2 partitions into membrane compartments and that its localization to the membrane allows phosphorylation of its specific substrates (Figure 4). mTORC2, along with its substrates, can also be found in non-membrane compartments but scaffolded by compartment-specific proteins.

mTORC2 localizes to and is active at the plasma membrane [131]. Its components, SIN1, and rictor, could contribute to its membrane localization. Three isoforms of SIN1 (α, β, δ) have a PH domain [39]. The PH domain is likely critical for both SIN1 and mTORC2 localization to membrane compartments as the SIN1 isoform that lacks a PH domain interacts with other mTORC2 components but does not localize to the plasma membrane [48,49,131] and is instead found in cytosolic structures [39]. Since the PH domain binds PIP3, whose levels are augmented during PI3K activation, it is possible that there is increased sequestration or assembly of mTORC2 at the plasma membrane upon enhanced growth factor/PI3K signaling. SIN1-PH domain mutations increase cellular oncogenicity by preventing mTOR inhibition, highlighting the role of this domain in mTORC2 activation [49]. In addition to PIP3 binding, the SIN1-PH also binds to several types of membrane phosphoinositides *in vitro* [132]. Furthermore, based on sequence analysis, rictor is also predicted to harbor PH domains and may thus also modulate mTORC2 membrane localization [38]. Notably, in *S. cerevisiae* the plasma membrane localization of TORC2 is not dependent on AVO1 PH domain or phospholipids (PIP2) but is instead influenced by AVO3 (rictor) [133].

mTORC2 localization to the membrane enhances phosphorylation and activation of its substrates that it compartmentalizes with. Increased growth factor/PI3K signaling elevates levels of PIP3, which attracts other signaling molecules such as those containing PH domains including the mTORC2 target, Akt [134,135]. At the membrane, PDK1, another AGC kinase family member that contains a PH domain, also binds PIP3 and enhances Akt phosphorylation (Thr308) at the catalytic loop, thus increasing Akt activity [136]. Membrane-bound Akt mutants are constitutively phosphorylated at Thr308 as well as Ser473 in the hydrophobic motif (HM), suggesting that PDK1 and mTORC2 are enriched at the membrane [137–139]. It is noteworthy that Akt could also autophosphorylate and is phosphorylated by other mTORC2-independent kinases in other contexts as discussed further below (see section ‘Akt’). Other targets of mTORC2 also contain distinct lipid-binding motifs that could mediate membrane localization and thus facilitate their phosphorylation by mTORC2 in a membrane compartment. Two major substrates of mTORC2, the conventional and novel PKC (cPKC and nPKC) and SGK3 both harbor lipid-binding motifs (see sections ‘SGK’ and ‘PKC’). Other lipid-anchored proteins may also mediate substrate phosphorylation by mTORC2. For example, in endothelial cells, PKCα localization to the plasma membrane recruits mTORC2 to rafts in a manner dependent on syndecan-4, a single-pass transmembrane protein that binds to PIP2 [140]. The latter allows syndecan-4 oligomerization and binding of PKCα.

mTORC2 also localizes to the endoplasmic reticulum (ER) periphery [141]. Growth factor signaling does not affect mTORC2 localization to this compartment. Although mTORC2 is also found associated with ribosomes [101,142], it remains unclear whether its localization to the ER is related to its function during translation. At the ER membrane, mTORC2 assembly and association with ribosomes is modulated by ER-leaky Ca^2+^ from the Ca^2+^ channel-like protein TMBIM6 (transmembrane B cell lymphoma 2-associated X protein inhibitor motif-containing 6) [143]. Regulation of mTORC2 by TMBIM6 stimulates anabolic metabolism. mTORC2 is also found near a region of the ER called MAM (mitochondria-associated ER membranes), which is linked to mitochondria [144]. Its localization to MAM occurs in a growth factor-dependent manner and enhances Akt and SGK1/NDRG1 signaling [144–146]. Activated Akt phosphorylates MAM-associated proteins including the inositol trisphosphate receptor (IP3R), hexokinase 2, and phosphofurin acidic cluster sorting protein 2 (PACS2). Whether mTORC2 localization to the ER and/or MAM is mediated by phosphoinositides is not clear. Its activation in this compartment is linked to its role in maintaining MAM integrity and control of calcium release at MAM, mitochondrial function, and interaction with the ribosome [144].

mTORC2 is also found in lysosomal membrane fractions. Pools of mTORC2 and Akt are sensitive to lysosomal positioning in the cell since perinuclear clustering of lysosomes delays their activation during serum replenishment [147]. mTORC2 is present and active on subpopulations of endosomes [131]. In this compartment, the scaffolding protein IQGAP1 (IQ-motif-containing GTPase-activating protein 1) assembles the PI3K/Akt pathway [148]. However, it is not clear if the activity and localization of mTORC2 in the endosomal pool subset is dependent on growth factors/PI3K. This mTORC2 pool could have enhanced activity via phosphorylation of SIN1 at Thr86, which further elevates Akt activity. Endosomal mTORC2/Akt could speculatively facilitate the trafficking of this signalosome to subcellular membrane compartments to phosphorylate a variety of Akt substrates.

mTORC2 exists in the perinuclear and nuclear compartments. mTORC2 phosphorylates the SGK1 HM site at a perinuclear compartment in response to angiotensin II (AngII) [127]. SIN1 and SGK1 also colocalize in this compartment in a cPKC-dependent manner, which modulates SIN1 phosphorylation. Following genotoxic stress, a signalosome consisting of p53, the nuclear PI3K inositol polyphosphate multikinase (IPMK), PIP3, Akt, PDK1, and mTORC2 is formed in the non-membranous nucleoplasm [149]. The activation of Akt in this compartment promotes the phosphorylation of FOXO, leading to the inhibition of DNA damage-induced apoptosis. Interestingly, this pathway is insensitive to conventional PI3K inhibitors. p53 serves as the scaffold for the nuclear IPMK-mTORC2-Akt signalosome, which is distinguished from the conventional class I PI3K-mTORC2-Akt complex that is scaffolded via IQGAP1 [149,150].

Further studies are needed to delineate the specific signals that control mTORC2 subcellular localization and the substrates that it regulates in various compartments.

## Activators of mTORC2

**Figure 5.**
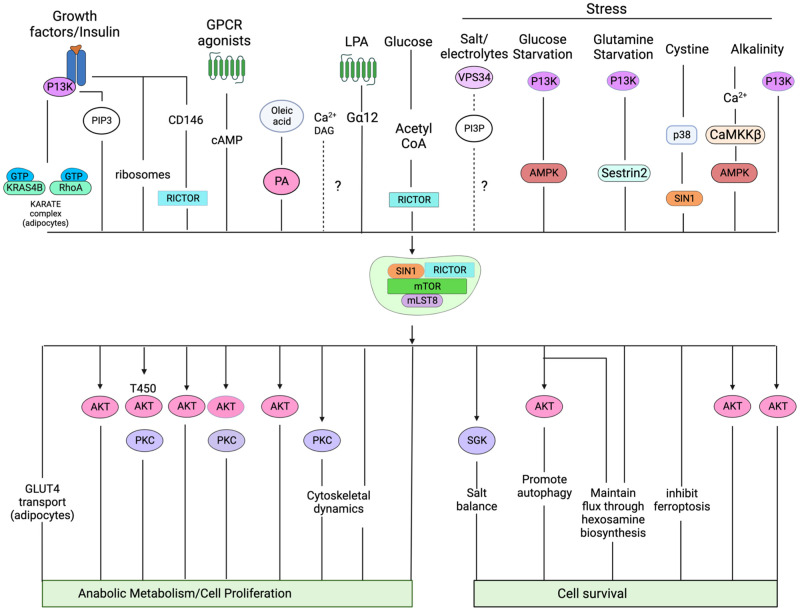
A variety of cellular signals contribute to mTORC2 activation. mTORC2 activators range from growth factors to nutrients to environmental stress. Signals from these stimuli are relayed to mTORC2 via other signaling proteins, phosphoinositides, lipids, and other secondary messengers. These stimuli could allow recruitment of mTORC2 substrates such as Akt, PKC, and SGK to cellular compartments where they colocalize with and are phosphorylated by mTORC2. How mTORC2 is regulated by each of these stimuli remains poorly understood. Shown are the stimuli (top) and their corresponding downstream effectors (bottom) that are phosphorylated or mediated by mTORC2 signaling. The cellular output of such signals includes increased anabolic metabolism/cell proliferation or enhanced cell survival.

mTORC2 is modulated by various signals including growth factors, hormones, nutrients, and stress conditions (Figure 5). As discussed above, the recruitment of mTORC2 to the surface of membrane-containing compartments and post-translational modifications of its components play crucial roles in modulating its activity. More recent studies also reveal that mTORC2 may be activated in non-membrane-containing locations. There is also an emerging role of G-protein coupled receptor (GPCR) signaling in regulating mTORC2.

### Insulin, growth factors, and PI3K

Signals from growth factors/hormones such as insulin modulate mTORC2 signaling. The binding of insulin to the insulin receptor (IR) promotes the autophosphorylation of the IR, leading to the recruitment of signaling molecules that bind to its phosphorylated residues [151]. One of the signaling molecules that is triggered by IR signaling is PI3K. PI3K, which generates PIP3 by phosphorylation of PtdIns(4,5)P2 (PIP2), is also activated by stimulation of other growth-related cell surface receptors. The intensity of receptor stimulation, and hence PI3K activation, could modulate mTORC2 signaling. For example, the increased generation of PIP3 during strong T cell receptor (TCR) stimulation enhances mTORC2 activation and phosphorylation of Akt at Ser473, whereas weak TCR signals only generate low PIP3 levels that are sufficient to phosphorylate Akt at the PDK1-mediated site, Thr308, but not mTORC2-targeted Ser473 [152]. As discussed above (see section ‘mTORC2 cellular localization’), one possible mechanism in PI3K modulation of mTORC2 is the binding of PIP3 to SIN1. This interaction between PIP3 and SIN1 occurs via the latter's PH domain and increases mTORC2 activity by releasing steric SIN1-PH inhibition on mTOR [49,153].

Increased growth factor/PI3K signaling also enhances mTORC2 activation and its association with ribosomes [142]. NIP7, a protein that is involved in rRNA maturation of the 60S ribosomal subunit, is involved in mTORC2 signaling in the ribosomal compartment. The function of mTORC2 in this compartment remains to be further examined. The finding that mTORC2 phosphorylates Akt-Thr450 during translation is consistent with the presence of active mTORC2 associated with ribosomes [101]. Although Akt-Thr450 phosphorylation is not sensitive to PI3K inhibitors [154], Akt translation itself may be enhanced by insulin/PI3K signaling and thus corroborate increased mTORC2 association with ribosomes during growth factor/PI3K signaling.

Insulin stimulation also promotes the assembly of a supercomplex containing KRAS4B-RhoA and mTORC2 (termed KARATE) [155]. KARATE controls the translocation of GLUT4 to the plasma membrane during insulin stimulation in adipocytes. How the GTPases KRAS4B and RhoA could activate mTORC2 activity remains unclear. The subcellular compartment where mTORC2 gets activated and the involvement of membrane lipids in this KARATE complex would also need to be further investigated.

Growth factors can influence mTORC2 activity independently of PI3K. After growth factor stimulation, the transmembrane cell adhesion protein CD146 protects the rictor from ubiquitination. The binding of rictor/mTORC2 to the phosphorylated intracellular domain of CD146 upon growth factor stimulation enhances mTORC2 activity, cell proliferation, and survival [120]. CD146 is necessary for the mTORC2-mediated Akt-Ser473 phosphorylation but not for PDK1-mediated Akt-Thr308 phosphorylation, suggesting that maintaining mTORC2 in this membrane compartment is sufficient for its activity. Precisely how sequestering mTORC2 with CD146 prevents rictor ubiquitination remains to be investigated.

While most studies have demonstrated the role of class I PI3K and its product PIP3 in mTORC2 regulation, it is conceivable that other lipid kinases and/or their products could modulate mTORC2. Class III PI3K, such as the family member Vps34 (vacuolar protein sorting 34) phosphorylates phosphatidylinositol (PtdIns) to produce PtdIns 3-phosphate (PI3P). PI3P is present in the endosomes where mTORC2 is located [131], and the generation of this phospholipid enhances SGK3 phosphorylation [156,157]. It is also noteworthy that in *S. cerevisiae* TORC2 signaling is modulated by PI4,5P2 [133], which is generated by the type I PtdIns4P 5-kinase MSS4 (multicopy suppressor of *stt4* mutation). How mTORC2 localizes to these lipid-containing compartments to phosphorylate its targets requires further inquiry.

### G-protein coupled receptor signaling

Accumulating evidence supports that mTORC2 is activated by GPCR signaling. β- and α-adrenoceptors increase cAMP levels and promote glucose uptake via mTORC2 but are independent of PI3K/Akt signaling. This mode of mTORC2 activation and modulation of glucose uptake has been observed in skeletal muscle, adipose tissue, and cardiomyocytes [158–160]. Epac1, a target protein of cAMP, may play a role in this mechanism as some studies have linked mTORC2 activation by adrenergic signaling via Epac1 [161,162]. In brown adipocytes, the β3-adrenergic signaling to promote glucose metabolism and thermogenesis is mediated by mTORC2–Akt [161]. The GPCR/cAMP signaling to mTORC2 is conserved in yeast. The *Schizzosaccharomyces pombe* GPCR, Git3, transduces signals to heterotrimeric G proteins in the presence of glucose. The GPCR/G proteins/PKA signaling activates TORC2, leading to Gad8 (Akt/SGK homolog) phosphorylation [163]. Hence, the GPCR regulation of mTORC2 is conserved from yeast to humans but the involvement of other AGC kinases in mediating mTORC2 function in mammals would need to be further interrogated.

mTORC2 could also couple glucagon receptor (GCGR), a GPCR-type receptor, and IR signals to enhance insulin sensitivity. Although long-term GCGR is linked to hyperglycemia and glucose intolerance (and thus antagonistic to insulin signaling), acute GCGR potentiates glucose metabolism and insulin sensitivity by a mechanism that involves hepatic rictor/mTORC2 and Akt signaling [164]. How mTORC2 could couple signals from these two receptors to enhance insulin signaling is unclear.

The G proteins that couple signals from GPCR to mTORC2 remain poorly understood. The heterotrimeric G proteins, consisting of GDP-loaded Gα that interacts with a Gβγ heterodimer, transduce signals from GPCR. Several isoforms of Gβγ could interact with the kinase domain of mTOR in a serum-inducible manner [165]. The Gα12 mediates signals from LPA, leading to mTORC2 activation to promote cell migration [166]. Future studies should reveal how G proteins could transduce signals to mTORC2.

Taken together, mTORC2 contributes to anabolic glucose metabolism when stimulated by GPCR signaling and this function could be uncoupled from insulin/PI3K/Akt signaling. Such a distinct mode for mTORC2 activation could have significant implications in improving glucose metabolism during insulin resistance and for therapeutic targeting of other diseases that have deregulated GPCR signaling.

### Amino acids and alkalinity

mTORC2 activation is affected by intracellular nutrients or metabolites. Unlike mTORC1 whose activity is positively correlated with nutrient levels, mTORC2 seems to respond to nutrient fluctuations, i.e. both increased or decreased levels. Early studies have shown that amino acid re-addition to serum- and amino acid-deprived cells increases mTORC2 phosphorylation of Akt at Ser473 [167]. Since pharmacological inhibition of PI3K prevents Akt phosphorylation during amino acid restimulation, this would suggest a requirement for PI3K during this response. It is unclear, however, how PI3K could mediate signals from amino acids. Recently, it was shown that alkaline pH enhances mTORC2 activity. Re-addition of an amino acid mixture that has an alkaline pH, but not if adjusted to physiological pH, increases Akt phosphorylation [168]. These findings imply that mTORC2 may not directly respond to increased amino acids but to the pH condition of the intracellular milieu. Addition of NH_4_Cl or NH_4_OH also activates mTORC2 signaling [169]. The increased intracellular alkalinity appears to involve mobilization of Ca^2+^ and activation of CaMKKβ [168,169]. The latter promotes the activation of AMPK, which consequently enhances mTORC2 presumably by phosphorylation of mTOR [170]. Increased alkalinity also activates PI3K by a mechanism that does not involve serum growth factors [171]. How mTORC2 could reprogram metabolism in response to changes in alkalinity and thus drive malignancy deserves further investigation. Other amino acids such as cystine activate mTORC2 via promoting SIN1 phosphorylation [172]. Cystine activates p38 MAPK and this MAPK regulates mTORC2 assembly and activation to prevent cancer cell ferroptosis. Future studies should address whether specific amino acids can promote mTORC2 activation under particular conditions or whether their effects on mTORC2/MAPK signaling may also be indirect. Amino acid sensors that could directly couple amino acid level signals to mTORC2, much like those identified for mTORC1 [173], would need to be elucidated.

Consistent with the role of mTORC2 in restoring metabolic homeostasis during nutrient fluctuations, mTORC2 is activated by glutamine deprivation [174]. This response requires PI3K. Akt-Ser473 phosphorylation also increases during cystine or leucine withdrawal under similar conditions, suggesting that mTORC2 may respond to the amino acid limitation in general [174]. The activation of mTORC2 during glutamine limitation allows the maintenance of flux through the hexosamine biosynthesis pathway (HBP), a metabolic pathway that uses glucose and glutamine for the biosynthesis of UDP-GlcNAc, which is used in protein and lipid glycosylation [174,175]. Glutamine catabolites, such as α-ketoglutarate, could be sensed by mTORC2 but the mechanisms remain obscure. It is worth noting that mTORC2 activation during nutrient withdrawal does not occur immediately but only after a few hours, suggesting a more adaptive response, likely to enhance cell survival. Sestrin2 could potentially mediate mTORC2's response to glutamine starvation. In glutamine-starved lung cancer cells, Sestrin2 associates with mTORC2, both of which are up-regulated, and repress mTORC1 activity to promote cell survival [176]. Sestrin2 also increases Akt phosphorylation through the Sestrin/GATOR2/mTORC2 axis [64]. In addition to Sestrin 2, Sesn3 also interacts with rictor to promote mTORC2 activation [177]. How Sestrins could mediate signals from glutamine level fluctuations remains to be further examined. In addition to glutamine starvation, Akt is also activated in response to deprivation of other amino acids including arginine, methionine, and lysine. The phosphorylation of Akt at Ser473 by mTORC2 is mediated by a signaling axis that involves GCN2, ATF4, and REDD1 [178]. This response occurs to promote cell survival during amino acid deprivation. The loss of REDD1 or mTORC2 components prevents Akt phosphorylation and further diminishes cell viability during glutamine limitation. Since ATF4 promotes the expression of REDD1 and Sestrin2 leading to mTORC1 inhibition during amino acid limitation [179], these findings reveal that GCN2/ATF4 signaling could positively signal to mTORC2 under these conditions. Precisely how this counter-regulation of mTORC1 versus mTORC2 occurs remains to be further investigated.

While mTORC2 is required to respond to changes in amino acid levels, it could serve as a checkpoint to control cell cycle progression or proliferation depending on nutrient availability. In support of this notion, loss of rictor in CD4^+^ T cells permits their proliferation despite arginine deficiency, a condition that usually arrests T cells in the G1 phase of the cell cycle [180]. Future studies should address how mTORC2 senses amino acid levels and its role in re-establishing metabolic homeostasis.

### Glucose

mTORC2, primarily via Akt regulation, plays a central role in glucose metabolism. Hence, it has a conserved role in responding to intracellular glucose level fluctuations [163]. Activation of mTORC2 during glucose starvation increases Akt phosphorylation at Ser473 [174]. The increased Akt phosphorylation during glucose starvation leads to phosphorylation and repression of GSK3β [181]. This repression of GSK3β promotes the nuclear retention of the transcription factor TFEB (transcription factor EB), which activates the expression of genes involved in autophagy and lysosome biogenesis during nutrient limitation, thus facilitating the mobilization of internal nutrient sources. Glucose starvation directly activates mTORC2 via AMPK phosphorylation of mTOR at Ser1261 and likely other sites in mTOR as well as rictor [170]. Phosphorylation of mTOR, as part of mTORC2, during glucose starvation requires PI3K signals. Interestingly, glucose deprivation attenuates another mTORC2-targeted site in Akt, Thr450 [126]. Since the phosphorylation of this site by mTORC2 occurs independently of PI3K [154], this would suggest that mTORC2 could be modulated by PI3K-dependent and -independent signals in response to glucose fluctuations. mTORC2 signals may also be indirectly up-regulated by AMPK during glucose starvation via AMPK regulation of mTORC1 [182]. Decreased mTORC1 signals augment PI3K signaling by a feedback mechanism and consequently enhance PI3K/mTORC2 signaling [183]. The activation of mTORC2 during glucose fluctuations enables flux through critical metabolic pathways that utilize glycolytic metabolites such as the HBP via modulation of the key enzyme of the HBP, GFAT1 [174,184]. mTORC2 likely modulates other metabolic pathways in response to glucose level fluctuations to re-establish metabolic homeostasis and promote cell survival [170,174].

Glucose availability also directly regulates mTORC2 via acetylation of rictor [116]. Rictor can be persistently acetylated in GBM cells leading to autoactivation of mTORC2. Elevated levels of acetyl-CoA, which is generated by the metabolism of glucose, glutamine, and/or fatty acids, occur in GBM and facilitate rictor acetylation. The increased mTORC2 signals render GBM cells resistant to inhibition of upstream signals such as growth factor receptor or PI3K signaling.

These findings indicate that the activity of mTORC2, via modifications of its components, is sensitive to glucose level fluctuations. How it regulates flux through metabolic pathways that require glucose metabolites, such as its role in modulating the HBP [174], deserves further investigation.

### Lipids/fatty acids/membrane tension

As discussed above, mTORC2 is modulated by increased levels of phosphoinositides upon growth factor signaling (see section ‘Insulin, growth factors, and PI3K’) but whether and how it responds to other lipids remains obscure. mTORC2 is activated by the class I PI3K-generated phosphoinositide, PIP3, via binding of PIP3 to SIN1 to allosterically modulate mTORC2 activity [49]. Another mTORC2 substrate SGK3 is phosphorylated upon binding of the latter to the class III PI3K-generated PI3P. Whether PI3P also interacts directly with mTORC2 components or if it enhances recruitment of mTORC2 to endosomes where PI3P is enriched remains to be investigated. mTORC2 controls various aspects of lipid metabolism [185–188]. In yeast, plasma membrane stress, such as by inhibition of sphingolipid metabolism, hypotonic stress, or membrane stretch, activates TORC2 signaling [187]. Some studies in mammalian cells have shown how specific lipids can activate mTORC2. The addition of fatty acids, in particular oleic acid, to serum-starved cells enhances mTORC2 (and mTORC1) activation [189]. In KRas-driven cancer cells that utilize serum lipids for their proliferation, the activation of mTORC2 by oleic acid depends on *de novo* synthesis of phosphatidic acid (PA) [189], a lipid metabolite that is used for phospholipid biosynthesis. The generation of PA requires the production of the glycolysis intermediate glycerol-3-phosphate and the synthesis of fatty acyl-CoA. PA has been previously shown to activate mTORC1 [190]. PA, which is generated via a phospholipase D-catalyzed reaction that converts phosphatidylcholine to choline and PA, binds to the FRB domain of mTOR and modulates the stability and activity of mTORC2 as well as mTORC1. It remains to be examined how the binding of specific lipids could modulate mTORC2 activity towards specific substrates, particularly those that promote lipid metabolism. It is also possible that lipids could bind to scaffolding proteins that serve as platforms for mTORC2 signaling.

## Targets and cellular functions of mTORC2

**Figure 6.**
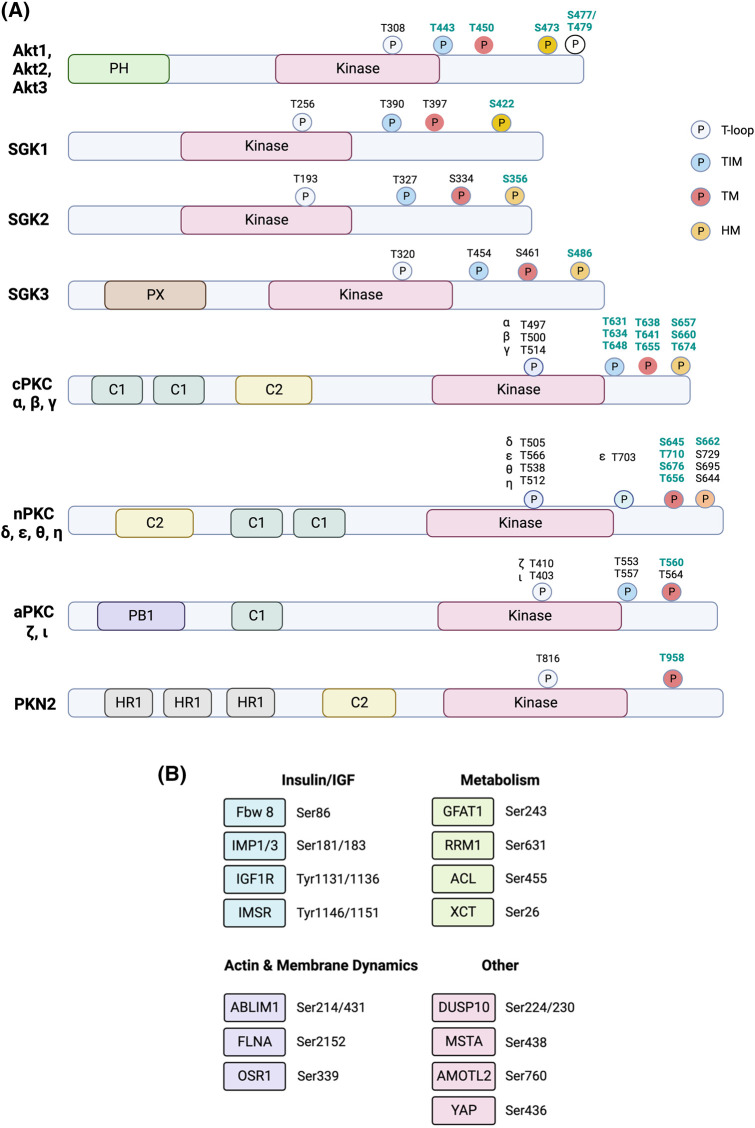
Key mTORC2 substrates and their phosphorylation sites. (**A**) mTORC2 phosphorylates Akt, SGK, and different PKC family members as well as the PKC-related PKN2 either directly or indirectly. Substrate domains and phosphorylation sites are indicated. Phosphorylation sites are specified as part of the TOR interaction motif (TIM), hydrophobic motif (HM), turn motif (TM), or T-loop. Sites that are phosphorylated by mTORC2 are in green fonts. (**B**) Additional mTORC2 substrates and phosphorylation sites, grouped broadly by cellular function.

The most well-documented mTORC2 substrates are members of the AGC (PKA, PKG, PKC) family of protein kinases that includes Akt/PKB, PKC, and SGK [191,192] (Figure 6A). These AGC kinases have a plethora of substrates that mediate their diverse cellular functions. Their catalytic activity is increased by phosphorylation at the activation loop (also called T-loop) by another AGC kinase, PDK1. PDK1, which is constitutively active, docks to the phosphorylated or acidic residues (in the case of atypical PKCs) of the HM of these AGC kinases to facilitate T-loop phosphorylation. mTORC2 mediates the phosphorylation of these AGC kinases at the C-terminal motifs that include the turn motif (TM). Recently, a TOR interaction motif (TIM), which is N-terminally adjacent to the TM has been identified to be targeted also by mTORC2, and phosphorylation at this motif could facilitate PKC and Akt HM site autophosphorylation [193]. mTORC2 phosphorylation promotes the proper folding and stability of the newly synthesized AGC protein kinases [154,194]. Inducible phosphorylation of the AGC kinases at allosteric sites, such as the HM of Akt, further enhances their catalytic activity. A variety of non-AGC kinase mTORC2 substrates, such as other signaling molecules, metabolic regulators, and transcriptional regulators have also been identified. So far, ∼26 substrates have been characterized and experimentally verified as mTORC2 substrates [195] (Figure 6B). Phosphorylation of some mTORC2 targets requires growth factor and PI3K signaling while others do not, suggesting that mTORC2 can phosphorylate substrates under different conditions, likely through cellular compartmentalization [127,131]. The precise mechanisms underlying mTORC2 localization and phosphorylation of its substrates in different compartments remain elusive. Although SIN1 could bind PIP3 via its PH domain and could thus enhance mTORC2 localization to membrane compartments, some studies indicate that SIN1 subcellular localization is not altered during growth factor stimulation or PI3K inhibition [127,131]. Whether mTORC2 assembly is enhanced in these compartments is not clear, however. Other signals, such as post-translational modifications, or association with compartment-specific scaffolds could promote mTORC2 localization to membrane compartments where it encounters and phosphorylates its targets.

### Akt

Akt is a Ser/Thr kinase that has a canonical role in regulating glucose metabolism [196]. Hence, it is often deregulated in cancer and diabetes. However, it is also involved in a variety of cellular functions such as during proliferation, cell survival, development, and other metabolic processes. It has three isoforms, Akt1, Akt2, and Akt3, that are encoded by different genes but share conserved structural motifs. Akt has a PH domain at its amino terminus that binds to the phospholipids PIP2 and PIP3, the latter leading to stronger activation of Akt [196]. The PIP3/PIP2 ratio could determine levels of Akt phosphorylation and consequently its activation state. Its catalytic activity is increased by phosphorylation at the T-loop site, Thr308, by PDK1. Akt is allosterically phosphorylated at other sites to further enhance its activity or stability. mTORC2 mediates phosphorylation at the TM, TIM, and the HM of Akt. The phosphorylated forms of Akt engage distinct substrates and thus affect cellular outcome [18,152,197].

mTORC2 mediates the phosphorylation of Akt at Ser473 of the HM in response to the presence of growth factors, which enhances PI3K activation [198,199]. The phosphorylation of this site is often used as a hallmark of mTORC2 activation, although there is a growing number of other protein kinases including Akt itself (by autophosphorylation) that have been shown to phosphorylate this site [140,200–209]. Interestingly, DNA-PKcs (DNA-dependent protein kinase catalytic subunit), which belongs to the same PIKK-related protein kinase family as mTOR, also associates with SIN1 and this association is necessary for Akt phosphorylation by this kinase [210,211]. The CRIM domain in SIN1 facilitates Akt phosphorylation at Ser473 [30,43,212]. Often, the ectopic expression of membrane-targeted Akt, which leads to constitutive Akt activation alleviates defects associated with lack of endogenous Akt phosphorylation (e.g. during Akt, mTORC2, or PDK1 disruption) [213,214], underscoring the role of membrane localization of Akt in facilitating its activation. The phosphorylation at Ser473 in Akt1 (Ser474 in Akt2, Ser472 in Akt3) further enhances Akt activity, enabling it to phosphorylate distinct and in most cases, more substrates [18,26,215–217]. Phosphorylation at Thr308 and/or Ser473 affects Akt function and ultimately, cell fate. Both cellular and *in vivo* studies provide evidence that the phosphorylation at the HM site can occur with [218,219] or without Thr308 phosphorylation [18,154,220]. This distinct regulation could be due to differences in cell type or stimulatory conditions. The phosphorylation at Ser473 could also prime active Akt for proteasomal degradation. Phosphorylation at this site promotes Lys48-linked polyubiquitination of Akt to destabilize Akt [221]. Such a mechanism could allow for the termination of Akt signals.

mTORC2 also phosphorylates Akt at Thr450 of the TM. Thr450 phosphorylation is constitutive, independent of PI3K, and occurs during Akt translation [101,154]. Phosphorylation at this site contributes to the structural stability of Akt by linking Akt's carboxyl tail to its kinase domain [154]. Phosphorylation at Thr450 is dependent on sufficient levels of glucose due to the sensitivity of mTORC2 to ATP levels [126]. However, a phosphomimetic mutant of SIN1 on Ser260 could allow Akt-Thr450 phosphorylation despite low glucose levels, indicating that glucose metabolism could modulate SIN1 post-translationally. The distinct regulation of Akt-Thr450 and Ser473 by mTORC2 suggests that their phosphorylation may occur in different cellular compartments.

mTORC2 phosphorylates a TIM, just distal to the kinase fold and has the consensus motif -FD-x-x-FT- that is present in Akt (and several mTOR-targeted PKC isoforms). Phosphorylation at this motif (Thr443 in Akt1) was proposed to facilitate subsequent phosphorylation at the T-loop (Thr308) by PDK1 and HM site (Ser473) by autophosphorylation [193]. Hence, these studies propose that mTORC2 does not directly phosphorylate the HM site. Given that phosphorylation of Akt-Ser473 is triggered under different cellular conditions and present in various membrane-containing compartments, it is conceivable that it is phosphorylated unexclusively by mTORC2. Nevertheless, embryonic or tissue-specific knockouts of rictor, SIN1, or mLST8 are often devoid of Akt-Ser473 phosphorylation, underscoring the requirement for mTORC2 in mediating Akt HM site phosphorylation *in vivo* [18,25,26,222–224].

In addition to TM and HM sites, Akt is phosphorylated at the C-terminal tail at Ser477/Thr479 [225]. mTORC2 mediates the phosphorylation of these sites in a growth factor-dependent manner and potentiates Akt-Ser473 phosphorylation. However, these sites are also targeted in a cell cycle-regulated manner by CDK2/cyclin A.

Akt may also play a role in the feedback control of mTORC2. Akt phosphorylates SIN1 at Thr86 but whether this phosphorylation augments or diminishes mTORC2 activity is controversial. This phosphorylation serves as a positive feedback to mTORC2, thereby enhancing its activity and further elevating the phosphorylation of Akt and likely other substrates [124]. There is also contradictory evidence presented that phosphorylation at this site, as well as SIN1-Thr398, dissociates SIN1 from mTORC2, thereby inactivating mTORC2 [51]. The reciprocal regulation between Akt and SIN1/mTORC2 would need to be further examined under different cellular contexts.

Akt recognizes a plethora of cellular substrates that have a consensus motif of RXRXX(S/T) [196]. Among the most well-characterized Akt substrates that have been linked to mTORC2 signaling and are often markers of mTORC2 pathway activation are the protein kinase GSK3β and the transcription factor FOXO1/3. The phosphorylation of these two proteins by Akt restrains their activity. In the case of GSK3β, Akt-mediated phosphorylation dampens GSK3β kinase activity. Phosphorylation of FOXO1/3 sequesters this transcription factor into the cytoplasm and suppresses its role in gene transcription.

### SGK

The serum- and glucocorticoid-regulated kinase (SGK) is closely related to Akt/PKB and has three paralogs, SGK1, SGK2, and SGK3 [226]. SGK augments the expression and activity of various transporters including the Na^+^/K^+^ ATPase, glucose and amino acid carriers in the plasma membrane. It also promotes the up-regulation of ion channels including Na^+^, Ca^2+^, K^+^, and Cl^−^ channels. Its expression, particularly SGK1, is tightly regulated transcriptionally and post-transcriptionally. SGK1 expression is enhanced by aldosterone, a key hormone for electrolyte regulation. SGK2 transcription is induced by β_3_ adrenergic signaling in adipose tissue [227]. SGK3 (also called as cytokine-independent survival kinase CISK) harbors an N-terminal phosphatidylinositol 3-phosphate (PI3P)-binding PX (phox homology) domain that mediates its interaction with the phosphoinositide, PI3P, at endosomal membranes [228]. SGK1 also has a truncated stretch of amino acids in the N-terminus that binds phospholipids *in vitro*, whereas SGK2 N-terminus does not contain a known interaction motif [229]. However, all three SGK paralogs are phosphorylated at the HM by mTORC2 and T-loop by PDK1. Little is known about the phosphorylation of the SGK TM. Mutation of the TM sites, Ser397 and Ser401, destabilizes the structure of SGK1 [230]. Ser401 is constitutively phosphorylated and unlike Akt, TM phosphorylation of SGK1 enhances its activation. Whether mTORC2 phosphorylates SGK TM phosphorylation is unclear but the increased expression of Akt augments SGK1 TM phosphorylation, suggesting that this may recruit mTORC2 to phosphorylate the TM. Consistent with the presence of a lipid-binding domain, SGK3 is modulated by both class I and class III PI3K (VPS34) [156]. Binding of the SGK3 PX domain with PI3P, which is produced by either VPS34 or via conversion of the class I PI3K product, PIP3, into PI3P [231], promotes SGK3 phosphorylation at the T-loop residue by PDK1 and at the C-terminal Ser422 HM residue by mTORC2 [157,232]. Moreover, SGK3 rapidly activates its target, the Na^+^/H^+^ exchanger 3 (NHE3), a key intestinal Na^+^ transporter upon glucocorticoid-induced colocalization in recycling endosomes, whereas SGK1 and SGK2 remain diffusely distributed [233]. These findings suggest that membrane colocalization of SGK3 with its activators, PDK1 and mTORC2, could facilitate rapid activation of SGK3 and phosphorylation of its substrates.

SGK1 binding to phosphoinositides may also facilitate its phosphorylation at conserved motifs [229]. Its activation is dependent on PI3K via PDK1 and mTORC2. The mTORC2-mediated phosphorylation at Ser422 facilitates the binding of the PIF pocket of PDK1 to this phosphosite, thus enhancing phosphorylation at Thr256 of the T-loop. So far, studies demonstrate that mTORC2 phosphorylates SGK1 at a perinuclear compartment [127]. Upon stimulation of angiotensin receptor AT1R, cPKC promotes SIN1 and SGK1 subcellular localization to this perinuclear region. The receptor tyrosine kinase (RTK)-independent AT1R activation that leads to SGK1 but not Akt phosphorylation may occur via GPCR signals that are localized to nucleus, endosomes or MAMs, and could underlie the specificity of mTORC2–SGK1 activation [127]. Further studies are needed to elucidate how GPCR as well as PI3K signaling could modulate SGK activation in various cellular compartments.

The mTORC2-dependent phosphorylation of SGK1 plays a critical role in electrolyte homeostasis in the kidney. Signals that enhance mTORC2 activity, such as insulin and IGF, promote Na^+^ reabsorption via SGK1-mediated activation of epithelial Na^+^ channel (ENaC). SGK2 and SGK3 potently stimulate ENaC activity [234] and are also involved in regulating the NHE [235]. mTORC2 also responds to K^+^ concentration on the basolateral (blood) side of principal cells and thereby promotes K^+^ excretion while enhancing Na^+^ reabsorption [236,237]. The activation of SGK1 by mTORC2 via K^+^ sensing is specific since phosphorylation of other mTORC2 substrates such as PKC and Akt is not dependent on this signal. How mTORC2 could sense K^+^ levels remains to be elucidated.

Other proteins that interact with mTORC2 enable specific SGK1 regulation. Protor knockout mice have diminished phosphorylation of SGK1 and its substrate NDRG1 (N-myc down-regulated gene 1) in the kidney [56]. The absence of Protor does not affect Akt and PKCα phosphorylation in this organ, suggesting that Protor is required for specific regulation of SGK1 in the kidney. The phosphorylation of SGK1 at Ser422 during muscle differentiation is mediated by the interaction of rictor with the heterogeneous nuclear ribonucleoprotein M (hnRNP M) [238]. The interaction of rictor with hnRNP M does not affect the phosphorylation of Akt and PKC. Hence, the interaction of mTORC2 with specific proteins could allow distinct regulation of its substrates.

In addition to phosphorylation, SGK expression is modulated in a rictor-dependent manner. The N-terminus of SGK1 contains a motif required for ubiquitination and degradation by the 26S proteasome. Rictor associates with Cullin-1, forming a functional E3 ubiquitin ligase to modulate SGK1 expression [239]. The loss of rictor or increased phosphorylation of rictor at Thr1135 prevents SGK1 ubiquitination, thus enhancing its protein levels. SGK1 has a propensity to unfold, hence it is dependent on Hsp90 chaperone complexes for stability [240]. SGK1 is also subject to persistent dephosphorylation by phosphatases PP5 and PP2A, consistent with low basal phosphorylation of SGK1. Genotoxic stress prevents its dephosphorylation by a mechanism that involves DNA-PK, which acts upstream of mTORC2 and is independent of PI3K.

SGK phosphorylates overlapping and non-overlapping substrates with Akt. They share the same substrate phosphorylation consensus motif of RXRSS(S/T) [226]. Hence, the inhibition of Akt in cancer cells could lead to drug resistance due to elevated SGK1 activity and phosphorylation of common substrates with Akt [241]. Due to the similarity in their kinase domain, other regulatory regions in these kinases would confer substrate specificity by binding to distinct regulators including membrane lipids. Recently, SGK1 has been reported to bind to sphingolipid C4-ceramide, which promotes PI3K/mTOR-independent autophosphorylation at the HM site leading to increased SGK1 activation and function in membrane trafficking of a mutant cystic fibrosis transmembrane conductance regulator (CFTR) [242]. As discussed above, other proteins that bind to SGK could also modulate its subcellular localization. Cell-type specific expression of these kinases along with their substrates would also affect their function. The N-myc downstream-regulated gene 1 (NDRG1) is specifically phosphorylated by SGK1 and is often used as a hallmark of SGK and mTORC2 activation [243,244]. SGK1, as well as Akt, phosphorylates the E3 ubiquitin ligase NEDD4 (neural precursor cell expressed developmentally down-regulated protein 4) to negatively regulate its activity [245,246]. In T helper cells, mTORC2/SGK1/NEDD4 axis promotes Th2 lineage differentiation and at the same time, the SGK1-mediated phosphorylation of GSK3β inhibits Th1 development [247]. How the different SGK paralogs mediate the various functions of mTORC2 await further investigation.

### PKC

The protein kinase (PKC) family members include the conventional (cPKC including isoforms α, β_I_, β_II_, γ), novel (nPKC including δ, ε, η, θ) and atypical (aPKC including ζ, ι/λ) [248]. Members of this family share a similar structure; the N-terminal portion consists of regulatory modules that have varying sensitivities to diacylglycerol (DAG) and/or Ca^2+^, the C-terminal region contains the kinase domain, and a hinge region that connects the N- and C-terminal regions [249]. Whereas cPKC and nPKC both bind DAG via the C1 domain, aPKC has a variant C1 domain that does not bind DAG or phorbol esters. nPKC and aPKC do not respond to Ca^2+^. Despite varying modes of regulation, the different family members are modulated by mTORC2. Whether the signals that activate PKC could play a role in modulating mTORC2 remains a possibility given that mTORC2 components can bind to different lipids and that this complex is regulated by calcium/calmodulin [132,189,250]. The TM and HM phosphorylation of PKC occurs in an mTORC2-dependent manner as disruption or pharmacological inhibition of mTORC2 abolishes their phosphorylation. In cPKC, they are required for cPKC maturation to stabilize cPKC [154,194]. The C1 and C2 domains of cPKC are linked to membrane-based activation. The C2 domain specifically has lysine-rich β-strands that bind to PIP2, which may mediate both PKC recruitment to and retention at the plasma membrane [251,252]. At a membrane-containing compartment, mTORC2 facilitates phosphorylation of cPKC at the TM and HM, which are both located at the C-terminal region of PKC [154,194]. The HM phosphorylation of PKC occurs via autophosphorylation and is indirectly modulated by mTORC2. Newly synthesized PKC exist as dimers and upon phosphorylation at the TIM site (Thr634 in PKCβII), mTORC2 promotes autophosphorylation at the HM [193]. Both cPKC TM and HM phosphorylation occur independently of PI3K. TM phosphorylation of PKC, like that of Akt, is constitutive and critical for proper folding of the kinase domain [101,154,194]. PKCβII phosphorylation at the TM by mTORC2 can also be induced via the interaction of PKCβII with actin and clathrin followed by plasma membrane translocation [253]. Other lipid-anchored proteins may also mediate PKC phosphorylation by mTORC2. For example, in endothelial cells, PKCα localization to the plasma membrane recruits mTORC2 to rafts in a manner dependent on syndecan-4, a single-pass transmembrane protein that binds to PIP2 [140]. The latter allows syndecan-4 oligomerization and binding of PKCα.

mTORC2 is also involved in the regulation of nPKC. During LPA stimulation of fibroblasts, mTORC2 is activated leading to HM phosphorylation of PKCδ [166]. This occurs during the late phase of LPA stimulation, and is mediated by Gα12, which promotes the degradation of PRR5L (Protor1/2). PRR5L acts to specifically prevent mTORC2-dependent PKCδ HM site phosphorylation. The phosphorylation of PKCδ by mTORC2 in these cells plays a role in fibroblast migration and pulmonary fibrosis development. mTORC2 also has increased interaction with PKCδ (as well as PKCα) in cardiomyocytes upon exercise [254]. This interaction is associated with increased HM phosphorylation of PKCδ (and PKCα) and such phosphorylation suppresses the apoptotic activity of PKCδ during exercise while enhancing the PKCα-mediated cell survival. The CRIM domain of SIN1 interacts with PKCε (as well as PKCα) and the disruption of this interaction prevents PKC phosphorylation at the TM and HM [43]. Congenital mutation in PKCε, E599K, which results in partial loss of kinase activity, results in a short-stature syndrome [255]. Interestingly, this mutation is also associated with defective mTORC2 activation and Akt phosphorylation, suggesting that PKC may also modulate mTORC2 signaling. Induction of TM phosphorylation of another nPKC, PKCθ, in CD4-T cells by costimulation of CD3 and CD28 is also dependent on mTORC2 [256]. Complementation with active PKCθ could revert the Th2 cell defect in the absence of rictor, suggesting that this PKC isoform is also modulated by mTORC2. Another novel PKC family member Protein kinase C eta (PRKCH or PKCη) associates with rictor and its phosphorylation at Ser642, which is close to the TM site (Ser656), is rictor-dependent [257]. This phosphorylation plays a role in rictor-induced mouse embryonic stem cell differentiation.

For the atypical PKCζ, both rictor and mTOR are necessary for phosphorylation, which contributes to molecular stability and activity [258]. The mTORC2-mediated phosphorylation of PKCζ at the TM site is constitutive and occurs during translation [259]. Despite the involvement of PKCζ in insulin signaling, its activity is insulin unresponsive and not dependent on PIP3 but could be via substrate scaffolding. mTORC2 activity control of PKCζ also regulates the actin cytoskeleton through Rho GTPases [258].

The TM phosphorylation of the protein kinase C-related PKN2 (aka PRK2) is also dependent on mTORC2 [260]. Phosphorylation and activation of PKN2 promotes the activation of mTORC1 via PKN2-mediated inhibition of the PI3KC2-β function in the production of phosphatidylinositol-3,4-bisphosphate.

The effectors of mTORC2/PKC signaling remain to be characterized. As discussed above, the different PKC isoforms play roles in cytoskeleton-related processes, insulin signaling, and cell differentiation. How different stimuli mobilize PKC isoforms based on their distinct regulatory domains and how mTORC2 may respond to such stimuli to allow PKC phosphorylation warrant further investigation.

### Substrates involved in insulin/IGF signaling

In addition to the phosphorylation of AGC kinases that transduce cellular signals from insulin stimulation, mTORC2 also phosphorylates various proteins involved in insulin and insulin-like growth factor (IGF) signaling to promote signaling through these pathways. mTORC2 phosphorylates Ser86 of the F-box protein, Fbw8 (Fbxw8), the substrate-targeting subunit of the cullin 7 (CUL7) E3 ligase complex, allowing for protein stability and the translocation of Fbw8 to the cytosol, thus mediating IRS-1 degradation [261]. Fbw8 expression also decreases as a result of mTORC2 inhibition. Ser86 of Fbw8 conforms to the Ser/Thr-Pro-Pro motif that is present in the TM of AGC kinases that are targeted by mTORC2. Since IRS-1 is also phosphorylated at sites that dampen insulin signaling, the phosphorylation of Fbw8 by mTORC2 is a feedback mechanism to enhance IRS-1 turnover and thereby stall the development of resistance to insulin stimulation.

mTORC2 phosphorylates the IGF2-mRNA binding proteins (IMP) IMP1 (at Ser181) and IMP3 (Ser183). This phosphorylation occurs cotranslationally and strongly augments the binding of IMP1 to the IGF2-leader 3 5′UTR, which enables IGF2-leader 3 mRNA translational initiation by internal ribosomal entry (IRES) [262]. Loss of this mTORC2-mediated phosphorylation impairs splicing and translation of IGF2-mRNA.

Functioning as a tyrosine kinase, mTORC2 phosphorylates IGF1R at Tyr1131 and Tyr1136, and InsR at Tyr1146 and Tyr1151 [263]. SIN1 associates with IRS and recruits mTORC2 to IGF1R/InsR to promote the Tyr phosphorylation of these receptors. mTORC2 phosphorylates Tyr residues that are part of the triple tyrosine cluster, which is involved in amplifying the activation of InsR/IGF1R. While these findings provide further support for mTORC2 as a key regulator of insulin/IGF signaling, the role of mTORC2 as a dual-specificity kinase (i.e. Ser/Thr and Tyr kinase) would need further verification.

### Regulation of metabolism

mTORC2 controls diverse metabolic processes, including carbohydrate, protein and lipid metabolism, to modulate anabolic growth. Increased mTORC2 signaling not only enhances nutrient uptake but also augments flux through several metabolic pathways in order to generate ATP and intermediates that are required for macromolecule synthesis. During glucose metabolism (glycolysis), mTORC2 controls the expression and/or activity of glycolytic enzymes such as hexokinase (HK), phosphoglucose isomerase (PG), and phosphofructokinase (PFK) via modulating the transcription factors HIF1α and/or MYC [264,265]. Akt activation also increases glycolytic rate by HIF1-mediated induction of glycolytic genes, regulation of epigenetic regulators of glycolytic genes, modulation of glycolytic enzymes and promoting glucose transport [266–271]. The role of mTORC2 in the TCA cycle/mitochondrial respiration is more controversial. Transformed cells that rely on mTORC2 have increased dependence on mitochondrial functions, suggesting a positive role for mTORC2 in mitochondrial respiration [272]. Akt is also linked to increased activity of the respiratory complexes I, III, and IV. Akt enhances activity of these complexes by phosphorylating mitochondrial resident proteins such as the ATP synthase subunit [273]. The rapid activation of Akt by mTORC2 at mitochondria-ER contact sites recruits hexokinase I (HK-I) to the voltage-dependent anion channel on mitochondria, which then promotes mitochondrial respiration in memory CD8^+^ T cells [145]. This metabolic reprogramming enables memory CD8^+^ T cells to rapidly acquire effector functions upon antigen activation. Studies in mice liver also reveal that mTORC2 stimulates mitochondrial fission and respiration in response to fasting or lipid availability [146]. This mitochondrial function of mTORC2 is mediated by phosphorylation of NDRG1 at Ser336 through SGK1. Phosphorylated NDRG1 is necessary for recruitment and binding to CDC42 at MAMs. The finding that mTORC2 is activated during fasting (which increases lipid catabolism) to enhance mitochondrial respiration seems paradoxical, given that it also responds to lipid availability. However, its response to starvation would be consistent with the role of mTORC2 in re-establishing metabolic homeostasis during nutrient fluctuations. In other settings, mTORC2 disruption stimulates mitochondrial respiration [144,274,275] but also decreases α-ketoglutarate levels [174,274]. Since the TCA cycle relies on anaplerotic substrates (e.g. pyruvate, glutamine, fatty acids) to regenerate intermediates particularly during increased biosynthesis [276] or during nutrient starvation, these findings suggest that mTORC2 also plays a role in TCA anaplerosis and could coordinate mitochondrial respiration with the availability of particular nutrients. For example, the availability and metabolism of a key anaplerotic substrate glutamine is dependent on MYC, which is regulated by mTORC2 [265,277]. mTORC2 also phosphorylates the cystine–glutamate antiporter xCT at Ser26 to suppress glutamate secretion in cancer [278]. This allows utilization of glutamate as a carbon source for TCA anaplerosis and a nitrogen source for protein and nucleotide biosynthesis.

mTORC2 also regulates flux through biosynthetic pathways via modulation of the expression and phosphorylation of key metabolic enzymes. mTORC2 promotes flux through *de novo* hexosamine biosynthesis by modulating the key enzyme of this pathway, GFAT1 (glutamine:fructose-6-phosphate amidotransferase 1) [174,175]. During nutrient stress or conditions that compromise protein folding/glycosylation, mTORC2 senses fluctuations in glutamine catabolites to modulate the expression of GFAT1 via the transcription factor Xbp1s. It also modulates GFAT1 phosphorylation at Ser243 to promote its expression during nutrient limitation. Disruption of mTORC2 or GFAT1 deregulates UDP-GlcNAc production and protein glycosylation, leading to aberrant TCR expression due to misprocessing and consequently, impairs thymocyte development [224,279]. Interestingly, in the absence of GFAT1, rictor expression is abolished [279], suggesting that mTORC2 is also regulated by hexosamines.

Several enzymes involved in the pentose phosphate pathway (PPP) and nucleotide biosynthesis are also modulated by mTORC2 signaling. Akt stabilizes the rate-limiting enzyme of the PPP, glucose-6-phosphate dehydrogenase (G6PD), by inhibition of the E3 ubiquitin ligase TRIM21 that acts on G6PD [280]. This promotes generation of ribose 5-phosphate, which in turn maintains Akt activity by preventing expression of PHLDA3 (PH-like domain family A member 3), a negative regulator of Akt. In cancer cells that have increased PI3K activation (e.g. PTEN mutation), the negative regulators TRIM21 (tripartite motif-containing protein 21) and PHLDA3 are suppressed, thus enhancing the PPP and cell proliferation. Akt also phosphorylates Thr382 of transketolase (TKT), a key enzyme of the non-oxidative PPP [281]. This phosphorylation enhances flux through this branch of the PPP to augment purine synthesis. In addition to promoting purine synthesis, Akt2 modulates *de novo* pyrimidine synthesis by phosphorylation of Ser1859 and Ser1406 of CAD (carbamoyl-phosphate synthetase 2, aspartate transcarbamoylase, and dihydroorotase), the key enzyme of this metabolic pathway [282]. The expression of Akt2 is up-regulated in liver cancer that is characterized with activating mutations in the β-catenin protein. The Akt2-mediated CAD regulation occurs independent of mTORC1/S6K but it is unclear if mTORC2 signaling mediates the increased Akt2 activation in these tumors. Another nucleotide synthesis-related enzyme modulated by mTORC2 is the ribonucleotide reductase (RNR). mTORC2 phosphorylates RNR, which catalyzes the production of dNTPs for DNA replication [283]. mTORC2 phosphorylation of RNR large subunit RRM1 at Ser631 enables its interaction with small subunit RRM2 to enhance RNR catalytic activity and efficient DNA replication. This mechanism confers resistance to gemcitabine in non-small cell lung cancer and can be targeted pharmacologically to enhance DNA damage and cytotoxicity from gemcitabine.

The role of mTORC2 in *de novo* lipogenesis (DNL) is multi-faceted. First, it modulates the DNL key enzyme, ATP-citrate lyase (ACL/ACLY). It mediates phosphorylation of ACL at Ser455 to promote DNL [284,285]. This phosphorylation could occur via Akt [286] but it has also been shown to be phosphorylated by mTORC2 *in vitro*. Regulation of ACL induces PPAR-γ in brown preadipocytes and increases ChREBP (carbohydrate response element binding protein) in mature brown adipocytes. In the latter cells, mTORC2 also enhances histone acetylation and expression of gluco-lipogenic genes via ACL. mTORC2 promotes the major transcriptional regulator of lipid metabolism, sterol regulatory element binding protein (SREBP). mTORC2, via Akt-dependent and -independent mechanisms, controls the stability as well as activity of SREBP-1c in liver and cancer cells [222,287,288]. Additionally, mTORC2, via Akt, enhances the cell surface translocation of transporters such as CD36 and FATP1 (fatty acid transport protein 1), to facilitate uptake of fatty acids [289]. In the organism, mTORC2 controls adipogenesis. In white adipose tissue (WAT), mTORC2 controls the expression of the lipogenic transcription factor ChREBPβ (carbohydrate response element binding protein β) [290]. Increased glucose flux that is independent of the mTORC2 role in Akt phosphorylation underlies the regulation of this transcription factor in mature adipocytes. In brown adipose tissue (BAT), mTORC2, via Akt, selectively controls ACL phosphorylation to promote BAT glucose-dependent lipogenesis and differentiation [285]. mTORC2 also prevents lipid catabolism. In brown adipocytes, it suppresses the SIRT6-mediated FoxO1 deacetylation [291]. This occurs via mTORC2 and SIRT6 interaction and is independent of Akt.

Other mTORC2-regulated AGC kinases such as PKC and SGK also regulate glucose metabolism [292–294], mitochondrial respiration [295,296] and lipid metabolism [244,297]. While Akt and SGK display some degree of promiscuity in substrate recognition, they also have distinct metabolic targets that remain to be elucidated [298]. In some cases, the activation of PKC isoforms such as cPKC and nPKC could antagonize the role of mTORC2/Akt. This could occur during increased accumulation of intracellular fatty acids that activate specific PKC isoforms while impairing insulin/mTORC2 signaling [299,300]. Despite the common regulation conferred by the canonical HM/TM motifs and the T-loop in these AGC kinases, the unique regulatory domains that distinguish them from each other contribute to their activity, substrate specificity, and cellular outcome. Defining how mTORC2 could modulate these AGC kinases in response to various stimuli leading to metabolic consequences deserves further investigation.

### mTORC2 targets in actin and membrane dynamics

The actin cytoskeleton is important for movement of subcellular components, membrane trafficking, establishment of polarity, morphogenesis, and cellular motility for multicellular organisms. Actin dynamics, i.e. assembly and disassembly, facilitate spatial control of intracellular processes and cell growth. The function of mTORC2 in controlling the actin cytoskeleton is conserved from yeast to humans [10,16,17]. Studies have linked mTORC2 to small GTPases such as RhoA and Rac1, which function in cytoskeletal remodeling [16,301]. The defects in cytoskeletal remodeling upon mTORC2 disruption are rescued by the expression of constitutively active alleles of RhoA or Rac1 [16]. There is likely dynamic or reciprocal regulation between mTORC2 and small GTPase signaling as several studies have placed mTORC2 both upstream and downstream of these GTPases (see also section ‘Cross-talk with Ras and other small GTPases’). mTORC2 could modulate proteins that are involved in actin-based cell adhesion and migration via AGC kinase-dependent and -independent mechanisms. In oncogenic KRAS(G12D)-expressing pancreatic acinar cells that develop acinar-to-ductal metaplasia (ADM), mTORC2 activates the actin-related protein 2/3 complex (Arp2/3) via enhancing Akt/Rac1 signaling [302]. The activation of Arp2/3 by mTORC2 (as well as mTORC1) promotes ADM, a phenotypic switch that is characterized by marked cytoskeletal rearrangements and is involved in pancreatic carcinogenesis. mTORC2 also controls phosphorylation of actin-binding proteins. Rictor mediates the phosphorylation of the actin-binding LIM protein 1 (ABLIM1) in hepatocellular carcinoma [303]. ABLIM1 binds to actin filaments and mediates actin polymerization. Rictor knockdown or dominant-negative mutations of ABLIM1 at Ser214 and Ser431, which are putative mTORC2/rictor target sites, inhibit cell migration and actin polymerization. In GBM cells, rictor associates with filamin A (FLNA), a large cytoplasmic actin-binding protein [304]. mTORC2 mediates the phosphorylation of FLNA at Ser2152 in both GBM and melanoma cells [304,305]. This phosphorylation enhances focal adhesion formation and cell migration in the latter cells [305]. mTORC2 also interacts with gelsolin (GSN) to modulate GBM cell migration [306]. This binding mediates the interaction of mTORC2 with other cytoskeletal proteins and mTORC2 membrane localization. The cytoskeleton-related functions of mTORC2 are likely coupled to its role in metabolism. Mutations in isocitrate dehydrogenase (IDH) in glioma alter gene sets involved in cell motility, chemotaxis and invasion via mechanisms that involve rictor and Rac1 signaling [307]. These functions of mTORC2 in cytoskeleton reorganization and endocytosis could facilitate enhanced nutrient acquisition in metastatic tumor cells.

Related to its role in cytoskeleton reorganization, mTORC2 responds to plasma membrane tension. During chemotactic stimulation in neutrophils, increased membrane tension limits actin nucleation via mTORC2 and phospholipase D2 (PLD2). Disruption of mTORC2 in these cells results in larger leading edges, elevated membrane tension and impaired chemotaxis [308,309]. While the tension-based inhibition of leading-edge signals (Rac, F-actin) is independent of mTORC2 kinase activity, the regulation of RhoA and myosin II-based contractility at the trailing edge relies on its kinase activity. In the latter, mechanical membrane stretch, along with biochemical stimulation that increases PIP3, synergistically activate mTORC2 in these cells. The components of mTORC2 signaling that facilitate cell polarity and motility await further investigation.

Plasma membrane tension also occurs during osmotic stress. mTORC2 responds to these hyper- or hypo-osmotic shocks through a functional feedback loop based on plasma membrane tension such that mTORC2 activity is reduced as a result of decreased plasma membrane tension (hyperosmotic stress) and increased with greater tension (hypo-osmotic stress). mTORC2 up-regulates the transport of secretory vesicles containing the lipid sphingomyelin [310]. mTORC2, via Rab35, promotes the degradation of the actin cortex by increasing PIP2 metabolism. This in turn facilitates the integration of sphingomyelin-loaded vesicles into the apical membrane, thus alleviating plasma membrane tension. Additionally, mTORC2 targets proteins that are involved the osmotic stress response. mTORC2 phosphorylates the oxidative stress-responsive 1 (OSR1) at Ser339, which mediates the osmotic stress response [311]. OSR1 and the STE20/SPS1-related proline–alanine-rich kinase (SPAK) modulate the solute carrier 12 family of cation-chloride cotransporters that are involved in ion homeostasis.

While the above findings support the role of mTORC2 in controlling the actin cytoskeleton and membrane dynamics, more studies are needed to define specific mTORC2 targets that mediate its functions. How mTORC2 is modulated by signals from the cytoskeleton and membrane compartments also requires further investigation.

### Autophagy

Autophagy is a process that degrades and recycles intracellular proteins and organelles. There are three subtypes namely macroautophagy, chaperone-mediated autophagy (CMA), and microautophagy, each distinct depending on substrate acquisition. Relative to mTORC1, the involvement of mTORC2 in modulating autophagy is poorly understood. mTORC2, via its downstream AGC kinase targets, can either positively or negatively regulate autophagy. In the periphery of lysosomes, mTORC2, via Akt, negatively regulates CMA [312]. Akt phosphorylates GFAP (glial fibrillary acidic protein), which is involved in the assembly and disassembly of the LAMP-2A translocation complex during CMA. Increased GFAP phosphorylation destabilizes the translocation complex and reduces CMA. During starvation, the Akt phosphatase PHLPP1 counteracts mTORC2 action by inactivating Akt, thus promoting CMA. Akt also negatively regulates macroautophagy by phosphorylation of beclin1 at Ser234 and Ser295, which then promotes its association with 14-3-3 and vimentin filaments to consequently inhibit autophagosome formation [313]. The mTORC2 substrate, SGK1, has also been implicated in modulating autophagy. In muscle, it inhibits autophagy via regulation of ULK1, which is involved in the initiation of autophagosome formation [314]. In contrast with SGK1, SGK2 promotes autophagy by phosphorylating the subunit V1H(ATP6V1H) of V-ATPase proton pump in epithelial ovarian cancer cells [315]. Inhibition of SGK2 impairs autophagic flux and sensitizes these cells to platinum drugs. mTORC2 is also required for autophagosome biogenesis [316]. mTORC2, via PKCα/β, mediates the signals from IGF1R to promote clathrin-dependent endocytosis, which is necessary for autophagosome precursor formation. However, PKCβ also mitigates autophagy by modulating mitochondrial homeostasis. It will be important to define the precise conditions that allow mTORC2 and its AGC targets to either positively or negatively regulate autophagy. Given the role of mTORC2 in responding to either the abundance or limitation of nutrients, the context of mTORC2 activation likely determines its function in autophagy.

## Cross-talk with other pathways

### Cross-talk with mTORC1

**Figure 7.**
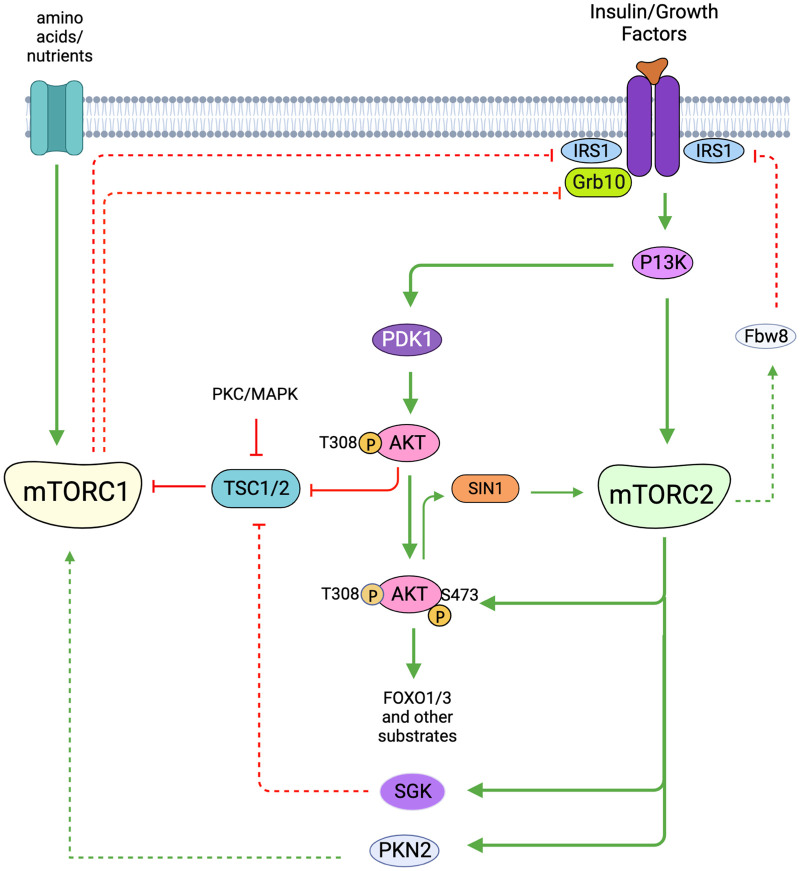
mTORC2 cross-talk with mTORC1. Feedback regulation occurs between mTORC2 and mTORC1 signaling to maintain metabolic homeostasis. Green solid lines indicate positive regulation and green dashed lines illustrate positive feedback. Red solid lines indicate negative regulation while red dashed lines depict negative feedback regulation.

As both mTOR complexes respond to nutrient levels and growth signals, there is extensive communication between the two mTORC pathways (Figure 7). Feedback signaling between these two complexes maintains metabolic homeostasis during nutrient fluctuations, avoids cell death during nutrient limitation and prevents deregulation of cell growth when nutrients are abundant. Increased mTORC1 signaling, such as during disruption of TSC1/2, feeds back to negatively modulate signals proximal to growth factor/PI3K signaling. It phosphorylates growth factor receptor-bound protein 10 (Grb10) to suppress insulin signaling and reduce mTORC2 activity [317]. Grb10 phosphorylation by mTORC1 promotes its accumulation and prevents PI3K signaling while Grb10 depletion enhances Akt phosphorylation. mTORC1 also modulates mTORC2 signaling via regulation of insulin receptor substrate-1 (IRS-1), a key transducer of insulin signals. Elevated mTORC1 signaling increases S6K1 activation, which feeds back to phosphorylate inhibitory sites on IRS-1 and curtails downstream mTORC2 activity, and consequently mitigates cell proliferation [318,319]. Diminishing mTORC1 signals via rapamycin treatment when TSC1/2 is disrupted relieves the feedback suppression of mTORC2 and could potentially hyperactivate mTORC2 [263,320]. While such treatment may promote malignancy, it could have benefits for neurological defects that have elevated mTORC1 signals [321,322]. Whether the derepression of mTORC2 signals in the latter could contribute to normalizing growth signals in TSC-deficient neurons would need to be investigated. It also remains undefined what other mTORC1-activating conditions other than loss of TSC function could trigger this negative feedback regulation.

While mTORC2 is modulated by feedback cues from mTORC1, mTORC2 also relays the presence of increased growth signals to mTORC1. Increased mTORC2/Akt signaling phosphorylates TSC2, which relieves mTORC1 inhibition by the TSC complex [323]. SGK1 and SGK3 also phosphorylates TSC2 independently of Akt [232,324]. PKC, either directly or indirectly via MAPK, also enhances TSC2 phosphorylation to promote mTORC1 signaling [325,326]. mTORC2 also enhances mTORC1 signaling via phosphorylation of PKN2 at the TM. PKN2 mediates mTORC1 activation by preventing the repression of mTORC1 by the phosphoinositide PIP2 at late endosomes and lysosomes [260]. A positive feedback loop also occurs as mTORC2 signaling is augmented. Increased PI3K signaling enhances Akt activation (by mTORC2-mediated Ser473 phosphorylation), which in turn further elevates mTORC2 signaling via Akt-mediated SIN1-Thr86 phosphorylation [124]. Elevated Akt phosphorylation then further amplifies mTORC1 signaling. Oncogenic mutations that lead to enhanced mTORC2 signaling (e.g. PTEN mutations) often have elevated mTORC1 signals and consequently increased anabolic metabolism and cell proliferation. mTORC2 also regulates the turnover of IRS-1 to modulate mTORC2/Akt signaling, which subsequently tunes mTORC1 signals. By restraining IRS-1 signaling, mTORC2 could thus down-regulate insulin and mTORC1 signaling [261]. Recently, it was reported that mTORC2 phosphorylates S6K1 at the HM site, which occurs after the autoinhibition of this kinase is first relieved by mTORC1 [327]. It is notable that while knockdown of rictor diminishes S6K1 HM phosphorylation [327], ablation of rictor or SIN1 using mouse models does not significantly affect S6K1 HM phosphorylation [18,25,26]. The conditions that trigger mTORC2 phosphorylation of S6K1 remain to be examined. Together these findings reveal how mTORC2 cross-talks with mTORC1 to modulate growth and proliferation in response to levels of nutrients and growth stimuli.

### Cross-talk with Ras and other small GTPases

mTORC2 is regulated by various small GTPases including Ras (Table 1). Whether this regulation occurs directly remains unclear. Consistent with the notion of more direct regulation of mTORC2 by Ras, there are RasGEFN and RBD in rictor and SIN1, respectively [48]. The SIN1-RBD can bind to HRAS, KRAS4A, KRAS4B, NRAS, and the binding of these Ras proteins to SIN1 is dependent on GTP [46,132]. Lower affinity binding may also occur with ERAS, RRAS, and RIT1 [132]. Mutations in the key residues of SIN1-RBD that mediate interaction with HRAS can occur in cancer, underscoring the role of this domain in SIN1 function [46,47,132]. Oncogenic Ras also directly binds to SIN1 as well as rictor [328,329]. This association depends on specific amino acids (T35, Y40, and E37) in the Ras effector domain [328]. Furthermore, disruption of the oncogenic Ras-mTORC2 interaction prevents Ras-dependent neoplasia *in vivo*. The role of the SIN1–Ras interaction in modulating mTORC2 activity and function is still contentious, however. In superoxide anion-stimulated breast cancer cells, disruption of Ras and SIN1 binding prevents mTORC2 activation and sequestration to the membrane [330]. In other cancer cell lines, the mutant oncogenic Ras associates with SIN1-RBD and that the GTP-bound mutant Ras increases mTORC2 kinase activity [331]. More recent studies challenge the role of Ras in mTORC2 regulation. SIN1 interacts mainly with the KRas4A isoform, via an atypical RBD (aRBD) [47]. Deletion of SIN1-aRBD does not diminish basal mTORC2 assembly or activity in HEK293T cells and in tissues of mice that express mutant SIN1-aRBD. These findings imply that SIN1–KRAS interaction may have mTORC2-independent functions. The reason for the discrepancies between these studies is unclear but may be explained by the use of cancer vs non-cancer models.

**Table 1.** Components of mTORC2/Ras cross-talk

Stimuli		1. Insulin			Insulin	Oxidative stress	EGF
		2. EGF					
		3. Superoxide anion (breast cancer cells)					
**Small GTPase**	Oncogenic Ras	HRas	KRas4A, KRas4B	Rac1	RhoA	Rit	Arf1
**Interaction**	SIN1/Ras	SIN1/HRas	SIN1/KRas4A	mTOR/Rac1	SIN1/RhoA	SIN1/Rit	Rictor/Arf1
	Rictor/Ras	*SIN1γ/HRas (*strong interaction)	SIN1/KRas4B		Rictor/XPLN		
**GEF**				P-Rex1	XPLN		
**Effect on mTOR or MAPK signaling**	Increased mTORC2 signaling	1. Inhibits ERK activation, enhances SGK1 activity	No effect on mTORC2 assembly or activity upon SIN1-aRBD mutation	Akt	Rictor/XPLN interaction inhibits mTORC2	Increased mTORC2 signaling	Decreased mTORC2 signaling
		2. No effect on ERK activation					
		3. Increased mTORC2 activation and membrane sequestration					
**Target**		1. SGK1 (NDRG1)		Akt (via Tiam1)/PKC (via inhibition of RhoGD12) potentiate Rac1 signals in a feedback loop	Akt	Akt	Akt
		2. ?					NDRG1
		3. Akt, PKCα					
**Function**	Neoplasia	1. ?		Cell migration	Apoptosis	Cell survival	Membrane trafficking
		2. ?					
		3. Cell migration/invasiveness					

List of known small GTPases that signal to mTORC2. The binding of small GTPases to a particular mTORC2 component is indicated. The cellular stimuli mediated by the mTORC2/small GTPase signaling are indicated if known. Participating guanine nucleotide exchange factors (GEF), effects on signaling, targets, and cellular function are also listed if known.

The Rho family GTPases are also linked to mTORC2 signaling (see also section ‘mTORC2 targets in actin and membrane dynamics’) [16]. Rac1 binds directly to mTOR and regulates the activity of both mTORC1 and mTORC2. The binding of Rac1 to mTOR is dependent on Rac1's CD but not its GTP-bound state [332]. The Rac1 GEF, P-Rex1, which interacts with both mTORC1 and mTORC2, could mediate Rac1 activation to promote cell migration [333]. In breast cancer, a positive feedback loop, based on mTORC2 targets Akt and PKC, potentiates Rac1 signals. In HER-2 breast cancer cells, Akt signaling stimulates Rac1 through Rac-GEF Tiam1, and PKC signaling reduces expression of a Rac1 inhibitor, RhoGD12 [334]. Consistent with the involvement of Rho GTPases in mTORC2 signaling, a Rho GTPase GEF, XPLN (Exchange factor found in platelets, leukemic, and neuronal tissues), also interacts with rictor to suppress mTORC2 activity but does not affect mTORC1 activity in HEK293 cells. XPLN inhibits Akt activity and its depletion prevents starvation-induced apoptosis in cells. This mechanism does not involve XPLN's exchange factor activity but does require an amino acid fragment in the molecule's N-terminus [335].

Another member of the Ras superfamily of GTPases, Rit, interacts with SIN1. In response to oxidative stress, Rit promotes cell survival via modulating mTORC2/Akt signaling [336]. The activation of Akt by Rit during oxidative stress is diminished by p38 inhibition. Whether the Rit/SIN1 interaction could serve to link the mTORC2 and p38 MAPK pathway to enhance cell survival remains to be further examined.

The ADP ribosylation factor 1 (ARF1), a member of the Ras superfamily of GTPases that are involved in vesicle trafficking, interacts with rictor [337]. ARF1 negatively regulates EGF-stimulated Akt-Ser473 phosphorylation and NDRG1-Thr346 phosphorylation, suggesting that this small GTPase attenuates mTORC2 signaling. Whether ARF1 modulates mTORC2 signaling during membrane trafficking or how mTORC2 could be involved in this process will need to be investigated.

### Cross-talk with MAPK pathway

The mTORC2 and the MAPK (mitogen-activated protein kinases) pathways have conserved functions in promoting cell survival during stress conditions. SIN1 was originally identified in yeast as a protein that interacts with the fission yeast Sty1 (or Spc1), which is a component of the stress-activated protein kinase (SAPK) pathway and orthologue of p38 MAPK in mammals [338]. In yeast, stress conditions (e.g. osmolarity, oxidative stress, cell wall stress) down-regulate TORC2 signaling in a manner that requires MAPK. In budding yeast, the MAPK Slt2 phosphorylates TORC2 components to down-regulate TORC2 activity [339], while in fission yeast, the Spc-Atf1 pathway triggers metabolic adaptations to allow recovery of TORC2 signaling [340]. In mammals, cross-talk between mTORC2 and different MAPK pathways occurs to modulate not only growth during favorable conditions but also cell survival under stress. The prototypical MAPK signaling cassette consists of an initial GTPase, which regulates a MAPK kinase kinase (MAPKKK), which in turn phosphorylates a MAPKK that then phosphorylates and activates the effector MAPK (Table 2) [341]. mTORC2 and MAPK pathways are both triggered by stimulation of growth factor receptors and GPCR. There is accumulating evidence on the cross-talk between these two pathways, occurring at multiple levels, leading to coregulation of each pathway in response to specific stimuli. Mammalian SIN1 interacts with MAPK signaling components [29,342]. SIN1 binds to Ras, which is part of the Ras/ERK signaling cascade, but the downstream effects are still controversial [48,343,344] (see section ‘Cross-talk with Ras and other small GTPases’). The binding affinity of Ras with the different RBD-containing proteins that include SIN1 could contribute to the outcome of receptor stimulation. Notably, the SIN1–Ras association inhibits the ERK pathway during insulin stimulation. Since Ras also binds to other effectors such as Raf and PI3K, it is possible that the SIN1–Ras binding could sequester Ras away from its other downstream effectors, thus preventing ERK activation. However, the SIN1–Ras binding does not affect EGF-ERK signaling [47]. The activation state of Ras is likewise another contributing factor to the strength of ERK and PI3K/mTOR signaling. Indeed, oncogenic Ras (mutant H-, K-, and N-Ras) associates with mTOR and SIN1 to promote mTORC2 activity at the plasma membrane [328]. mTORC2 and ERK signaling also converge to modulate the AGC kinase RSK (p90 ribosomal S6 kinase). RSK contains two catalytic domains wherein one is homologous to AGC kinase domain (termed NTKD) and another to the calcium/calmodulin-dependent protein kinase (CaMK) (termed CTKD) family. Whereas ERK mediates the phosphorylation and activation of RSK, mTORC2 is required for optimal RSK HM site phosphorylation [345]. mTORC2 could act as a scaffold to allow RSK phosphorylation in response to nutrient levels and promote phosphorylation of specific RSK substrates such as the CCT complex.

**Table 2.** Components of mTORC2/MAPK cross-talk

Stimuli	Insulin	EGF		Amino acid cystine	
**GTPase**	Ras/SIN1	Ras/SIN1	Oncogenic Ras/mTOR/SIN1	Rit/SIN1	
**MAPKKK**	Raf	Raf		MEKK3	MEKK2/SIN1
**MAPKK**				MKK3/6	JNKK2
**MAPK**	ERK	ERK		p38	JNK1, ERK5
**Effect**	Ras/SIN1 interaction inhibits ERK	Ras/SIN1 binding does not affect EGF-ERK signaling	Increases mTORC2 activity, Modulates Rsk	Enhances mTORC2 activation, prevents cancer cell ferroptosis; mTORC2 inhibits DUSP10 to decrease p38 signaling as part of feedback loop	mTORC2 negatively regulates JNK pathway

The binding of mTORC2 signaling component(s) to a small GTPase and/or mitogen-activated protein kinase (MAPK) signaling component modulates MAPK and/or mTORC2 signaling in response to stimuli (indicated is known).

SIN1 also interacts with the small GTPase Rit, which is involved in the p38 MAPK cascade. During oxidative stress, Rit mediates the mTORC2-dependent phosphorylation of Akt to promote cell survival [336]. p38 is activated by the presence of the amino acid cystine [172]. Activated p38 mediates the phosphorylation of SIN1 to enhance mTORC2 activation. This response to cystine prevents cancer cell ferroptosis. In macrophages, the p38 pathway modulates the availability of PIP3 at the membrane during Toll-like receptor (TLR) stimulation, thereby positively regulating Akt phosphorylation [346]. Together, these findings suggest that p38 and mTORC2 are activated during stress conditions to promote cell survival. mTORC2 signaling also modulates p38, likely as a feedback regulation. In the absence of rictor in the liver, p38 signaling is attenuated, indicating that mTORC2 reciprocally modulates this MAPK pathway [347]. mTORC2 also phosphorylates the dual-specificity phosphatase DUSP10 at Ser224 and Ser230, leading to protein stability and decreased p38 signaling [348]. SGK1 phosphorylates MEKK3 at an AGC kinase substrate consensus motif and this phosphorylation inhibits MEKK3–MKK3/6 signaling [349]. Despite several studies that suggest a relationship between p38 MAPK and mTORC2 signaling, there is little known about the precise mechanisms that link these two pathways and their involvement in different types of cell stress.

SIN1's role as a MEKK2 and JNK interactor suggests cross-talk between the mTORC2 and JNK signaling cassette [29,342]. SIN1 (MIP1) interacts with the inactive and non-phosphorylated MEKK2, preventing its dimerization and activation [29]. Inactive MEKK2 stalls downstream signaling involving JNKK2, JNK1, and ERK5, suggesting that mTORC2 negatively regulates this MAPK pathway. Consistent with this notion, overexpression of SIN1 inhibits JNK activation by UV [342]. Furthermore, inhibition of mTORC2 increases JNK activation, without affecting ERK, in cultured HNSCC (head and neck squamous cell carcinoma cell) lines [350]. In these cells, JNK activation is required for cell death and its coinhibition with mTOR diminishes the effect of mTOR inhibitors, supporting the antagonistic relationship between mTORC2 and JNK signaling. However, the relationship is probably more complex since it has also been shown that JNK promotes mTORC2 signaling. In mesenchymal cells (MCs), JNK prevents the proteasomal degradation of SIN1 during LPA stimulation, thus promoting mTORC2 activation [351]. The JNK/mTORC2 signaling is required during the fibrotic activation of MCs. More studies are needed to define how mTORC2 is linked to JNK signaling and how their signals are coupled in response to specific cellular stimuli.

The atypical MAPKs are not activated by the classical three-tiered kinase cascades and their mode of activation is divergent from the conventional MAPKs. However, they modulate the mTORC2 target, Akt. MAPK4 and MAPK6, interact with Akt and phosphorylate Akt at Ser473 independently of mTORC2 [208,352]. Inhibiting MAPK6 sensitizes cancer cells to mTOR kinase inhibitors. Increased MAPK4 levels promote tumor progression while decreasing their levels inhibits cancer cell proliferation. Hence, combined inhibition of mTORC2 and atypical MAPKs could be necessary for more effective therapy of cancers that up-regulate these two signaling pathways.

### Cross-talk with Hippo/YAP pathway

**Figure 8.**
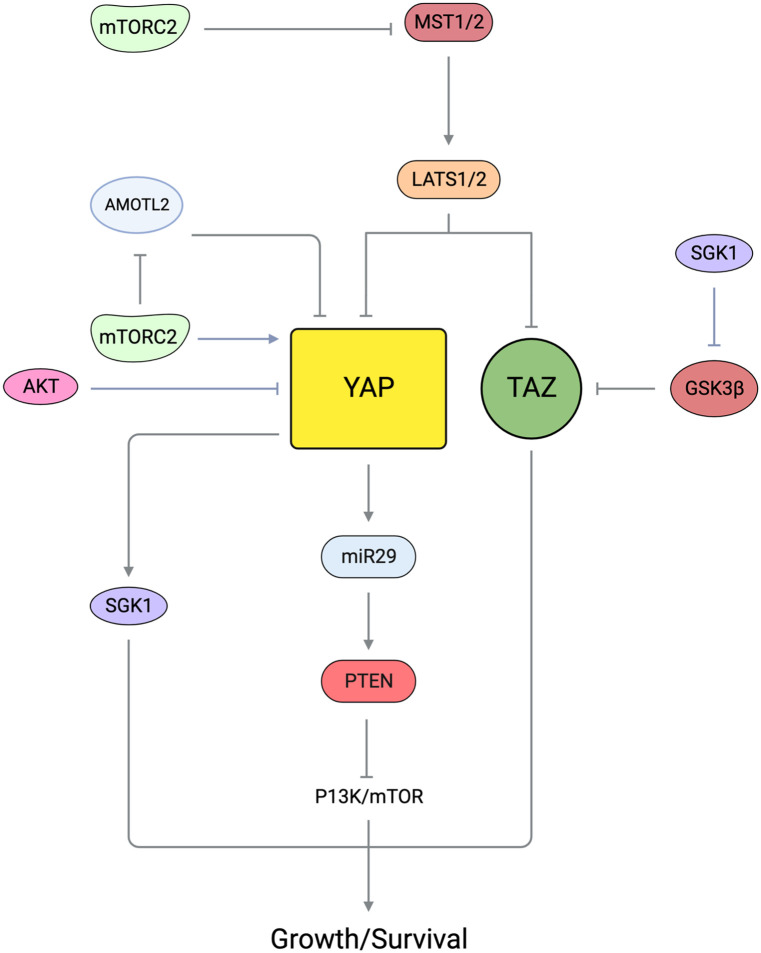
Various points of mTORC2 cross-talk with the Hippo/YAP pathway that affect cellular growth and survival. YAP and TAZ are transcriptional regulators that control growth and survival. Whereas mTORC2 promotes their activation at different levels including negative regulation of MST1/2 and AMOTL2 and phosphorylation of YAP.

Another signaling pathway that is involved in controlling cell growth and proliferation is the Hippo signaling cascade. Whereas the TOR pathway is highly conserved from yeast to human, the Hippo/YAP pathway originated in metazoans [353], befitting their known function in organ size control. The mTOR and Hippo pathways have antagonistic functions related to coordinating growth control (Figure 8). The mammalian homologs of the *Drosophila* Hippo, MST1/2 (mammalian Ste20-like kinases 1/2) phosphorylate the large tumor suppressor 1/2 (LATS1/2), which in turn phosphorylates and inhibits the transcriptional activators Yes-associated protein (YAP) and transcriptional coactivator with PDZ-binding motif (TAZ). Loss of inhibition of YAP/TAZ deregulates growth and often occurs in cancer [354]. mTORC2 phosphorylates MST1 at Ser438 and suppresses its activation by blocking homodimerization. In the heart, the function of mTORC2 in regulating MST1 is critical for promoting cardiac cell survival and growth [355]. Disruption of mTORC2 activates MST1, leading to cardiomyocyte apoptosis and cardiac dysfunction in response to stress. mTORC2 and MST1 signaling also modulate metabolism distinctly, thus affecting cell fate. MST1 promotes the development of invariant natural killer T cells (iNKT), favoring iNKT1 over iNKT17 development by promoting quiescence-associated metabolism [356]. During MST1 loss, mTORC2 signaling is enhanced to promote anabolic metabolism and iNKT17 development.

Cell growth is further supported by mTORC2 via negative regulation of the Hippo pathway protein, angiomotin 2 (AMOTL2) at Ser760 [357]. AMOTL2 is a negative upstream YAP regulator. Phosphorylation at this site prevents AMOTL binding to YAP, thus relieving YAP inhibition and promoting the growth of GBM cells. mTORC2 also phosphorylates YAP directly at Ser436 [358]. SIN1 recruits YAP to facilitate its phosphorylation by mTORC2. Phosphorylation at Ser436 increases YAP stability and promotes nuclear localization and transactivation of YAP-targeted genes. It also enhances GBM growth invasiveness [358]. Akt phosphorylates YAP as Ser127 to facilitate its cytoplasmic retention by binding to 14-3-3 proteins, consequently preventing apoptosis following DNA damage [359]. The mTORC2 substrate SGK1, by phosphorylating GSK3β, also modulates TAZ by preventing its ubiquitin-mediated degradation [360]. mTORC2 activity is linked to YAP/TAZ activation. In kidney fibroblasts, TGFβ1, which induces fibroblast activation and promotes kidney fibrosis, stimulates mTORC2 and YAP/TAZ activation [361]. Reciprocally, YAP also up-regulates mTORC2 signaling via PTEN [362]. YAP induces miR-29, which attenuates PTEN expression, and consequently triggers PI3K/mTOR activation to promote growth and proliferation. YAP/TAZ also directly activates SGK1 transcription [360]. Given the involvement of mTOR and YAP in promoting cell growth, inhibitors of these proteins have been used and shown to have promising therapeutic effects on TNBC models [363]. Future studies should reveal the extensive cross-talk and compensatory mechanisms between these two pathways to coordinate cell growth with tissue/organ growth.

## Targeting the mTORC2 pathway

**Figure 9.**
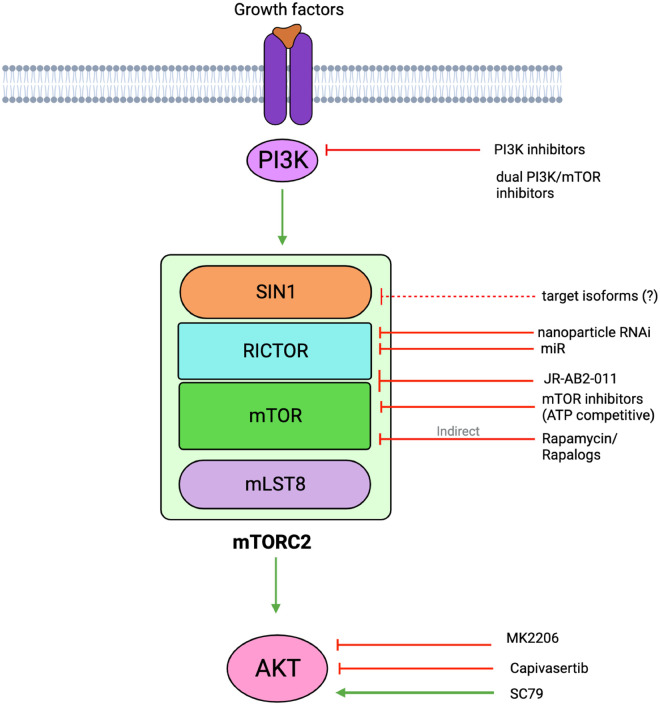
Targeting the mTORC2 pathway. Signaling molecules involved in mTORC2 signaling are potential targets to either dampen or enhance mTORC2 signaling. There is currently no specific mTORC2 inhibitor approved for clinical use.

The deregulation of mTORC2 signaling occurs in various diseases including cancer, diabetes, cardiovascular, immune-related, and neurodegenerative/neurological disorders. Hence, there are numerous efforts to alter the activity of this pathway, mostly via small molecule inhibitors. Rapamycin analogs, which selectively and allosterically inhibit mTORC1 but also indirectly alter mTORC2 activity, are currently the only approved mTOR inhibitors that are used in the clinic [8]. There are also mTOR inhibitors that block the catalytic activity of this protein kinase to prevent both mTORC1 and mTORC2 activity (Figure 9). Early results from clinical trials indicate that they may have limited use in the clinic, at least as monotherapy, due to toxicities [9]. However, biomarkers that can predict response to dual mTORC1/mTORC2 inhibitors may facilitate patient stratification and thus improve efficacy. Hyperactivating mutations in mTOR are found in various cancers and could indicate whether patients would benefit from mTOR inhibitors [364–369]. Another potentially useful biomarker is rictor expression. Its amplification in lung cancer cells correlates with sensitivity to mTORC1/mTORC2 inhibitors, leading to tumor stabilization in this subset of patients [370]. Many efforts are geared towards mTORC2 inhibition in cancer since rictor and SIN1 are often found overexpressed and mTORC2 downstream effectors such as Akt are up-regulated in different types of cancer. A compound that disrupts rictor-mTOR interaction (JR-AB2-011) displays cytotoxic and anti-metastasis effects in melanoma [329,371]. More *in vivo* studies are needed to verify the effects of this compound on mTORC2. Since many studies support that rictor expression is modulated at the mRNA level, the use of tissue-specific miRNA to target its expression could have benefits for cancer and other diseases that rely on up-regulated rictor expression for survival. Nanoparticle-based RNAi have been engineered and shown to decrease rictor expression and Akt phosphorylation, thereby increasing HER2-amplified tumor cell death [372]. The combination of the rictor RNAi with the HER2 inhibitor lapatinib *in vivo* also decreases the growth of HER2-amplified breast cancers more effectively than either treatment alone. It also selectively inhibits mTORC2 in TNBC, leading to decreased tumor growth. RNA-based therapy to target mTORC2 is, therefore, promising and deserves further development. Due to the presence of different SIN1 isoforms, targeting particular isoforms may have more specific effects and thus fewer undesirable outcomes. However, little is known about the functions and cell-type specificities of these isoforms. Disruption of SIN1 isoforms that interact with Ras may also impair Ras/MAPK signaling as has been shown in cancer cells that express oncogenic Ras [328]. This could be exploited to sensitize cancer cells that have gained resistance to Ras/MAPK inhibitors.

Dual PI3K and mTOR inhibitors have been developed for more effective blockade of downstream PI3K/mTORC2 effectors including the AGC kinases. However, results from clinical trials in different types of cancers suggest that these compounds have considerable toxicity [373–375]. Combining rapalogs with inhibitors of MAPK pathways has also been explored in clinical trials but seems to also have significant toxicity and limited efficacy [376–378]. To mitigate the undesirable side effects of mTOR inhibitors, drugs that target mTORC2 substrates are also being considered. Several Akt inhibitors have been developed and are undergoing clinical trials. When used as monotherapy in Phase II trials Akt inhibitor MK-2206 has limited activity despite the selection of patients with PTEN loss/mutations or constitutive PI3K/Akt activation [379]. However, when the Akt inhibitor capivasertib was added to fulvestrant, it extended survival of patients with aromatase inhibitor-resistant ER-positive, HER2-negative, advanced breast cancer and with PI3K/Akt/PTEN-altered tumors [380]. Sensitivities to Akt inhibitors could also depend on the degree and mechanisms of activation of the oncogenic mutant Akt [381]. Inhibitors that target different SGK and PKC isozymes are also being developed. Some of these drugs have undergone clinical trials but so far displayed poor efficacy [382,383]. Hence, while targeting downstream mTORC2 effectors may present with fewer toxicities, a better understanding of the aberrant molecular pathways as well as metabolic idiosyncrasies in patient tumors is needed to devise more effective strategies.

Aside from cancer, targeting rictor expression can also be effective in other diseases. micro-RNAs that down-regulate the expression of mTORC2 components could also be promising to treat diseases with increased mTORC2 signaling. Rictor is up-regulated in renal tissues of mice with diabetic nephropathy (DN) [384]. Increased expression of miR-424, which directly targets rictor expression, diminishes mTORC2 signaling and relieves the symptoms in the DN rat models. Targeting rictor with the miR-342-3p is also a key mechanism of action of glucocorticoids (dexamethasone) on regulatory T cells (Treg) to control inflammation [88]. Targeting mTORC2 is also promising for the treatment of several mTORopathies, which are neurological disorders characterized by aberrant mTOR signaling [385].

In some instances, it is possible that the use of rapalogs and mTOR inhibitors could have adverse effects due to suppression of mTORC2 functions. As discussed in this review, mTORC2 is crucial during stress responses and plays a role in re-establishing metabolic homeostasis. Rapalogs are used as therapy for tuberous sclerosis, LAM, some types of cancers and are also used as immunosuppressants. Chronic rapamycin exposure diminishes mTORC2 activity leading to increased insulin resistance and other metabolic consequences. It is, therefore, crucial to develop strategies that could maintain or sustain mTORC2 signaling during mTORC1 inhibition. Drugs that target specific mTORC1 signaling modulators may be useful for this. For example, the Rheb inhibitor NR1 only inhibits mTORC1 but not mTORC2 signaling [386]. Targeting specific mTORC1 components, such as raptor, or mTORC1 targets such as S6K, could be viable strategies [321,387]. Finding effective dosing, treatment regimens, and the development of more tailored mTOR inhibitors will also improve efficacy of these drugs. Last but not least, mTORC2 activators are also being developed and shown to improve treatment strategies for diseases of the central nervous system that utilize rapalogs and mTORC1 inhibitors [388–390]. Akt activators, such as SC79, may also show promise to enhance downstream mTORC2 signaling, which could alleviate metabolic defects and improve cell survival [391–394].

## Conclusion and perspectives

mTORC2 functions to control various aspects of metabolism. How mTORC2 responds to nutrient and other environmental fluctuations remains to be further interrogated. mTORC2 is activated by growth factors and PI3K signaling but it is also triggered by non-PI3K-dependent mechanisms that need to be further elucidated. Its involvement in GPCR signaling warrants further scrutiny as these receptors serve as important pharmacological targets for a variety of diseases. Developing better therapeutic strategies to manipulate mTORC2 signaling would require a more detailed understanding of how it modulates its AGC kinase substrates and other targets. In particular, how mTORC2 substrates are phosphorylated and mobilized in different cell types in a tissue microenvironment warrants further investigation as these mechanisms could offer important pharmacological targets for a variety of disease processes. The promiscuity of AGC kinases towards different substrates would need to be considered to achieve more effective inhibition of their functions in metabolism and cell growth. A better understanding of cross-talks with different growth-regulatory and stress-response pathways will also reveal more therapeutic opportunities. The control of rictor expression by specific miRNA in different cell types/tissues is also mounting and could be exploited for therapeutic purposes. The role of other non-coding RNAs such as lncRNA as well as RNA-binding proteins in mTORC2 signaling should be further explored. Much of the path forward lies in elucidating the selective modulation of mTORC2. By understanding the mechanisms and signaling pathways that can restrictively enhance or inhibit mTORC2, therapies can be developed to treat disorders that have deregulated metabolism while avoiding aberrant cell proliferation.

## Data Availability

There are no supporting data associated with this article.

## Abbreviations

4EBP1: eukaryotic translation initiation factor 4E-binding protein 1
ABLIM1: actin-binding LIM protein
Acetyl-CoA: acetyl coenzyme A
ACLY: ATP-citrate lyase
AD: armadillo domain
ADM: acinar-to-ductal metaplasia
AGC: protein kinase A (PKA), PKG, PKC
Akt/PKB: protein kinase B
AMOTL: angiomotin like 2
AMPK: AMP-activated protein kinase
AngII: angiotensin II
aRBD: atypical Ras-binding domain
ARF1: ADP ribosylation factor 1
Arp2/3: actin-related protein 2/3 complex
A-site: aminoacyl site
AT1R: AngII type 1 receptor
ATF1: G1 arrest defective
ATP: adenosine triphosphate
ATP6V1H: ATPase H+ transporting V1 subunit H
AUF1: ARE/poly(U)-binding/degradation factor 1
BAT: brown adipose tissue
BRCT: BRCA1 C-terminal
CAD: carbamoyl-phosphate synthetase 2, Aspartate transcarbamoylase, and dihydroorotase
CaMK: calcium/calmodulin-dependent protein kinase
CAMKKβ: calcium/calmodulin-dependent protein kinase kinase beta
cAMP: cyclic adenosine monophosphate
CASC9-1: cancer susceptibility candidate 9
CCT: chaperonin-containing TCP-1
CD: C-terminal domain
CD146: cluster of differentiation 146
CD8^+^: cytotoxic T cells
CDC: cell division cycle
CDK1/9: cyclin-dependent kinase 1/9
CFTR: cystic fibrosis transmembrane conductance regulator
ChREBP: carbohydrate response element binding protein
CISK: cytokine-independent survival kinase
CMA: chaperone-mediated autophagy
cPKC: conventional protein kinase C
CRIM: conserved region in the middle
Cryo-EM: cryogenic electron microscopy
CSCC: cervical squamous cell carcinoma
CTKD: C-terminal kinase domain
DAG: diacylglycerol
DEP: Dishevelled, Eg1-10, Pleckstrin
DEPTOR: DEP domain-containing mTOR-interacting protein
DN: diabetic nephropathy
DNA-PKc: DNA-dependent protein kinase catalytic subunit
DNL: *de novo* lipogenesis
dsRNA: double-stranded RNA
DUSP10: dual-specificity phosphatase 10
ECT2: epithelial cell transforming 2
EGFR: epidermal growth factor receptor signaling
Epac1: exchange protein directly activated by cAMP 1
ER: endoplasmic reticulum
ERAS: embryonic stem cell-expressed Ras
ERK: extracellular signal-related kinase
ESCC: esophageal squamous cell carcinoma
F-actin: filamentous actin
FAT: FRAP, ATM, TRRAP
FATC: FRAP, ATM, TRRAP C-terminal
FATP1: fatty acid transport protein 1
FBW7/FBXW7: F-box and WD repeat domain containing 7
FKBP: FK506-binding protein
FKBP12: 12-kDa FK506-binding protein
FLNA: filamin A
FOXO1/3: forkhead box protein O1/3
FRAP: FKBP12–rapamycin associated protein
FRB: FKBP12/rapamycin binding
G6PD: glucose-6-phosphate dehydrogenase
Gad8: G1 arrest defective 8
GATOR2: GAP activity towards Rags 2
GBM: glioblastoma
GCGR: glucagon receptor
GCN5L1: general control of amino acid synthesis 5 like 1
GDP: guanosine diphosphate
GEF: guanine nucleotide exchange factor
GFAP: glial fibrillary acidic protein
GFAT1: glutamine: fructose-6-phosphate amidotransferase 1
GLUT4: glucose transporter type 4
GPCR: G-protein coupled receptor
Grb10: growth factor receptor-bound protein 10
GSK3: glycogen synthase kinase 3
GSN: gelsolin
HBP: hexosamine biosynthesis pathway
HD: HEAT-like domain
HDAC: histone deacetylase
HEAT: huntingtin, elongation factor 3, A subunit of PP2A, TOR
HER2: human epidermal growth factor receptor 2
HIF1: hypoxia-inducible factor 1
HK-I: hexokinase I
hnRNP M: heterogeneous nuclear ribonucleoprotein M
hnRNPA2B1: heterogeneous nuclear ribonucleoprotein A2/B1
HNSCC: head and neck squamous cell carcinoma cell
HRAS: Harvey rat sarcoma virus
HSF1: heat shock factor 1
Hsp90/Hsp70: heat shock protein 90/70
HuR: human antigen R
IDH: isocitrate dehydrogenase
IGF: insulin-like growth factor
IGF1R: insulin-like growth factor 1 receptor
ILK: integrin-linked kinase
IMP: IGF2-mRNA binding proteins
iNKT: invariant natural killer T cell
InsP6: inositol hexakisphosphate
IP3R: inositol trisphosphate receptor
IPMK: inositol polyphosphate multikinase
IQGAP1: IQ-motif-containing GTPase-activating protein 1
IR: insulin receptor
IRES: internal ribosome entry site
IRS: Insulin receptor substrate
JNK: c-Jun N-terminal kinase
KARATE: KRAS4B-RhoA and mTORC2
KRAS4A/B: Kirsten rat sarcoma isoform 4A/4B
LAM: lymphangioleiomyomatosis
LAMP-2A: lysosome-associated membrane protein type 2A
LARP1: La ribonucleoprotein 1
LINC01133: long intergenic non-protein coding RNA 1133
LPA: lysophosphatidic acid
MAM: mitochondria-associated endoplasmic reticulum membranes
MAPK: mitogen-activated protein kinase
MAPKAP1: mitogen-activated protein kinase 2-associated protein 1
MAPKKK: MAPK kinase kinase
MEF: murine embryonic fibroblast
MEKK2/3: mitogen-activated protein kinase kinase kinase 2/3
MIP1: MEKK2-interacting protein 1
miR/miRNA: Micro-RNA
mLST8: mammalian lethal with sec13 protein 8
mRNA: messenger RNA
MS/MS: tandem mass spectrometry
MSS4: multicopy suppressor of *stt4* mutation
MST1/2: mammalian Ste20-like kinases 1/2
mTORC1/mTORC2: mammalian target of rapamycin complex 1/2
MYC: myelocytomatosis oncogene
MZF1: myeloid zinc finger 1
NAD+: nicotinamide adenine dinucleotide
ncRNA: non-coding RNA
NDRG1: N-myc downstream-regulated gene 1
NEDD4: neural precursor cell expressed developmentally down-regulated protein 4
NF-κB: nuclear factor kappa-light-chain-enhancer of activated B
NHE3: Na^+^/H^+^ exchanger 3
NIP7: nucleolar pre-rRNA processing protein NIP7
NK: natural killer cell
nPKC: novel PKC
NRAS: neuroblastoma Ras
Nrf2: nuclear factor erythroid 2-related factor 2
NTKD: N-terminal kinase domain
OSR1: oxidative stress-responsive 1
OTUD7B: OUT deubiquitinase 7B
p53: tumor protein P53
PA: phosphatidic acid
Pdcd4: programmed cell death 4 protein
PDK1: 3-phosphoinositide-dependent kinase 1
PDZ: PSD95, DlgA, Zo-1
PFK: phosphofructokinase
PG: phosphoglucose isomerase
PH: pleckstrin homology
PHLDA3: pleckstrin homology-like domain family A member 3
PHLPP1: PH domain leucine-rich repeat protein phosphatase 1
PI3K: phosphatidylinositol 3-kinase
PI3P: phosphatidylinositol 3-phosphate
PIK3C2β: phosphatidylinositol-4-phosphate-3-kinase catalytic subunit type 2 beta
PIKK: P13K-related kinase
PIP2: phosphatidylinositol 4,5-bisphosphate
PIP3: phosphatidylinositol-(3,4,5)-trisphosphate
PKC: protein kinase C
PKD: proteinase kinase D
PKN2: protein kinase N2
PLD2: phospholipase D2
PP2A: protein phosphatase 2A
PP5: protein phosphatase 5
PPAR-γ: peroxisome proliferator-activated receptor gamma
PPP: pentose phosphate pathway
PRK2: protein kinase C-related PKN2
PRKCH: protein kinase C eta
Protor1/2: protein observed with Rictor 1/2
PRR5: proline rich 5
PtdIns: phosphatidylinositol
PTEN: phosphatase and TENsin homolog deleted on chromosome 10
PX: phox homology
Rac1: Ras-related C3 botulinum toxin substrate 1
RAFT1: rapamycin and FKBP12 target 1
RAPT1: rapamycin target protein 1
Ras: rat sarcoma
RBD: Ras-binding domain
RhoA: Ras homolog family member A
Rictor: rapamycin-insensitive companion of mammalian target of rapamycin
RIT1: Ras-like protein expressed in many tissues
RNA: ribonucleic acid
RNAi: RNA interface
RNR: ribonucleotide reductase
ROS: reactive oxygen species
rpS6: ribosomal protein S6
RRAS: RAS related
RRM1/2: ribonucleotide reductase catalytic subunit M1/M2
RSK: ribosomal S6 kinase
RTK: receptor tyrosine kinase
S6K: S6 kinase
SAPK: stress-activated protein kinase
SGK: serum/glucocorticoid-regulated kinase
SIN1: stress-activated protein kinase-interacting protein 1
SIRT1/6: sirtuin 1/6
SLT2: suppressor of the Ly/Tic phenotype
SMN: survival motor neuron
SPAK: SPS1-related proline/alanine-rich kinase
SPC1: suppressor of Phosphatase 2C
SREBP: sterol regulatory element binding protein
STAT3: signal transducer and activator of transcription 3
STE20: Sterile 20
STY1: suppressor of tyrosine phosphatese
TAZ: transcriptional coactivator with PDZ-binding motif
TBK1: TANK-binding kinase 1
TCA: tricarboxylic acid cycle
TCR: T cell receptor
TFEB: transcription factor EB
TIM: TOR interaction motif
TLR: Toll-like receptor
TM: turn motif
TMBIM6: transmembrane B cell lymphoma 2-associated X protein inhibitor motif-containing 6
TNBC: triple-negative breast cancer
TNFα: tumor necrosis factor alpha
TOR1/TOR2: target of rapamycin 1/2
TRAF2: tumor necrosis factor receptor associated factor-2
Treg: regulatory T cell
TRIM21: tripartite motif-containing protein 21
TSC1/2: tuberous sclerosis complex 1/2
TTP: tristetraprolin
TTT: Tel2–Tti1–Tti1
UDP-GlcNAc: uridine-5′-diphospho-*N*-acetylglucosamine
USP: ubiquitin-specific protease
UTR: untranslated region
VPS34: vacuolar protein sorting 34
WAT: white adipose tissue
WDR24/WDR59: WD repeat-containing protein 24/59
xCT: cystine/glutamate transporter
XPLN: exchange factor found in platelets
YAP: Yes-associated protein
